# ZEB2 haploinsufficient Mowat-Wilson syndrome induced pluripotent stem cells show disrupted GABAergic transcriptional regulation and function

**DOI:** 10.3389/fnmol.2022.988993

**Published:** 2022-10-24

**Authors:** Jens Schuster, Joakim Klar, Ayda Khalfallah, Loora Laan, Jan Hoeber, Ambrin Fatima, Velin Marita Sequeira, Zhe Jin, Sergiy V. Korol, Mikael Huss, Ann Nordgren, Britt Marie Anderlid, Caroline Gallant, Bryndis Birnir, Niklas Dahl

**Affiliations:** ^1^Department of Immunology, Genetics and Pathology, Uppsala University and Science for Life Laboratory, Uppsala, Sweden; ^2^Department of Medical Cell Biology, Uppsala University, Uppsala, Sweden; ^3^Wallenberg Long-Term Bioinformatics Support, Science for Life Laboratory, Department of Biochemistry and Biophysics, Stockholm University, Stockholm, Sweden; ^4^Department of Molecular Medicine and Surgery, Center for Molecular Medicine, Karolinska Institutet, Stockholm, Sweden; ^5^Department of Clinical Genetics, Karolinska University Hospital, Stockholm, Sweden

**Keywords:** ZEB2, Mowat-Wilson syndrome, FOXG1, epilepsy, neurodevelopmental disease, GABAergic interneurons, transcriptional network, electrophysiology

## Abstract

Mowat-Wilson syndrome (MWS) is a severe neurodevelopmental disorder caused by heterozygous variants in the gene encoding transcription factor *ZEB2*. Affected individuals present with structural brain abnormalities, speech delay and epilepsy. In mice, conditional loss of Zeb2 causes hippocampal degeneration, altered migration and differentiation of GABAergic interneurons, a heterogeneous population of mainly inhibitory neurons of importance for maintaining normal excitability. To get insights into GABAergic development and function in MWS we investigated ZEB2 haploinsufficient induced pluripotent stem cells (iPSC) of MWS subjects together with iPSC of healthy donors. Analysis of RNA-sequencing data at two time points of GABAergic development revealed an attenuated interneuronal identity in MWS subject derived iPSC with enrichment of differentially expressed genes required for transcriptional regulation, cell fate transition and forebrain patterning. The ZEB2 haploinsufficient neural stem cells (NSCs) showed downregulation of genes required for ventral telencephalon specification, such as FOXG1, accompanied by an impaired migratory capacity. Further differentiation into GABAergic interneuronal cells uncovered upregulation of transcription factors promoting pallial and excitatory neurons whereas cortical markers were downregulated. The differentially expressed genes formed a neural protein-protein network with extensive connections to well-established epilepsy genes. Analysis of electrophysiological properties in ZEB2 haploinsufficient GABAergic cells revealed overt perturbations manifested as impaired firing of repeated action potentials. Our iPSC model of ZEB2 haploinsufficient GABAergic development thus uncovers a dysregulated gene network leading to immature interneurons with mixed identity and altered electrophysiological properties, suggesting mechanisms contributing to the neuropathogenesis and seizures in MWS.

## Introduction

Mowat-Wilson syndrome (MWS) is a rare disease characterized by intellectual disability (ID), speech impairment, epilepsy and Hirschsprung disease (Mowat et al., 1998; Adam et al., 2006; Garavelli and Mainardi, 2007; Ivanovski et al., 2018). Mowat-Wilson syndrome is usually caused by heterozygous *de novo* variants in the *ZEB2* gene encoding the zink-finger E-box binding homeobox (ZEB) 2 transcription factor (Zweier et al., 2002). Epilepsy is one predominant feature in MWS and 80–90% of cases present with either focal, absence or generalized seizures (Garavelli et al., 2017; Ivanovski et al., 2018). Brain imaging of affected individuals has detected structural changes in a majority of cases, predominantly localized to the ventricular temporal horn and hippocampus (Garavelli et al., 2017).

The transcription factor (TF) ZEB2 is a key regulator throughout nervous system development and it is expressed in the neural tube, neural crest cells, hippocampus and the cerebral cortex (Chng et al., 2010; McKinsey et al., 2013; Brinkmann and Quintes, 2017). Mice deficient of Zeb2 (*Zeb2*−/−) die at embryonic day (E) E9.5 with failed closure of the neural tube (Van de Putte et al., 2003) whereas heterozygous mice (*Zeb2*−/+) survive with a reduced number of cortical interneurons and without seizures (Takagi et al., 2015). Conditional neural loss of Zeb2 leads to reduced size of the hippocampus (Miquelajauregui et al., 2007) and immature cortical identity of GABAergic interneurons (van den Berghe et al., 2013) accompanied by increased amounts of GABAergic neurons in the striatum, suggesting a defective migration (McKinsey et al., 2013). While these previous animal studies have brought essential information on the role of Zeb2 for development of GABAergic interneurons, the effects of ZEB2 haploinsufficiency on human GABAergic development and function in MWS remain unclear.

GABAergic interneurons comprise a heterogeneous and mainly inhibitory cell population that is subclassified by transcriptomic signatures (Huang and Paul, 2019). The diversity of interneurons enables a variety of inhibitory control mechanisms on cerebral microcircuits (Hensch, 2005; Haider et al., 2006) to balance network activity as well as to prevent runaway excitation of the neocortex and hippocampus (Kepecs and Fishell, 2014; Paz and Huguenard, 2015; Gouwens et al., 2020). Accordingly, deficient GABAergic activity has been implicated in the etiology of hyperexcitability and seizures (Galanopoulou, 2010; Khoshkhoo et al., 2017; Wang Y. et al., 2017; Pfisterer et al., 2020).

Herein, we used ZEB2 haploinsufficient subject derived induced pluripotent stem cell (iPSC) to model the transcriptional profile and function of GABAergic interneurons in MWS. Our model uncovered a network of co-expressed and differentially expressed genes (DEGs) accompanied by deficient migration in NSCs and altered electrophysiological properties of GABAergic neurons, providing insights into the neuropathogenesis and mechanisms underlying seizures in MWS.

## Materials and methods

### Subjects

Two full siblings (MW1 and MW2) with typical clinical features of MWS were included in the study. Patient MW1 is a boy delivered in Iraq after a normal pregnancy and he is the first child of healthy non-related parents. After moving to Sweden, the boy was diagnosed with neurodevelopmental delay, facial dysmorphisms, microcephaly, sensorineural hearing loss, hypospadias, retentio testis, aortic stenosis and Mb Hirschsprung. He had his first generalized seizure at age 5 years and walked independently at age 6 years. At age 15 years, he presented with facial features characteristic for MWS, moderate intellectual disability and he has been seizure free since the age of 12 years.

Patient MW2 is a girl delivered in Sweden at full term with generalized growth retardation (birth weight −3SD, body length −2SD, and head circumference −2SD). Corpus callosum agenesis was identified by ultrasound during pregnancy and confirmed after birth. Postnatal investigation revealed patent ductus arteriosus and pulmonary stenosis that resolved spontaneously. Growth retardation remained at 2 years of age (weight −1.5 SD, body length −2SD, head circumference −4SD) and she developed facial characteristics of MWS. She walked independently at age 2.5 years and had onset of febrile convulsions at the same age. Epilepsy developed later with partial and generalized seizures. The girl has myopia, strabismus, severe intellectual disability, no speech and sleep disorder.

**FIGURE 1:**
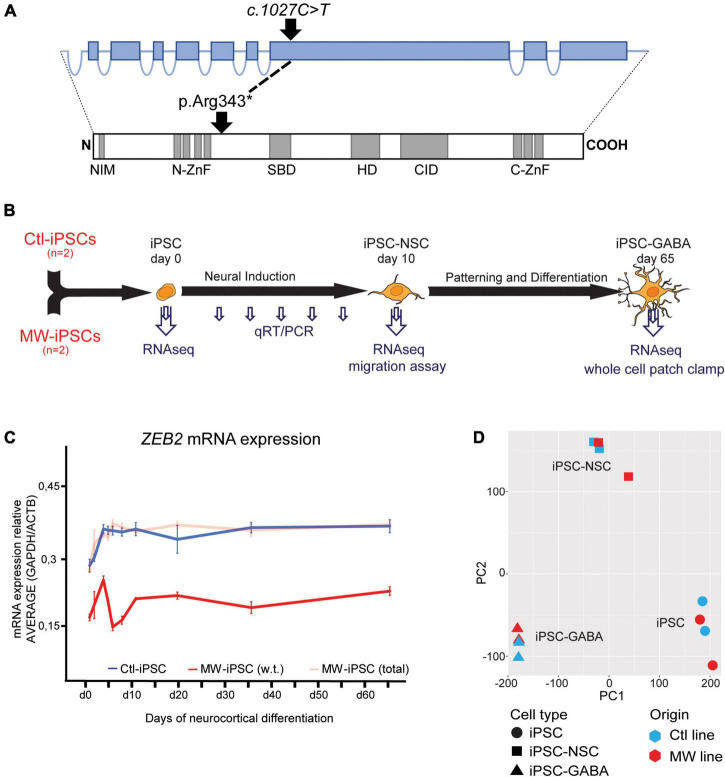
**(A)** Schematic illustration of the *ZEB2* gene structure with relative sizes of coding exons (top, blue boxes) and the corresponding ZEB2 protein architecture (bottom). The positions of the *ZEB2* gene variant and the resulting predicted stop-codon are shown with relation to functional protein domains (gray boxes). NIM, NuRD interacting motif; N-ZnF, N-terminal zink-finger clusters; SBD, Smad-binding domain; HD, Homeodomain-like domain; CID, CtBP-interacting domain; C-ZnF, C-terminal zink-finger clusters (modified from Birkhoff et al., 2021). **(B)** Schematic presentation of the iPSC differentiation procedure and time points of analysis using different methodologies. Induced pluripotent stem cells (iPSC) from two healthy donors (Ctl-iPSCs) and two MWS patients (MW-iPSCs) were harvested for qRT-PCR at d 0, 3, 4, 5, 7, 10, 19, 35, and 65 (small arrows) and for RNA-sequencing at days 0, 10 and 65 (large arrows). Migratory capacity was assessed on confluent iPSC-NSCs at d 10 and electrophysiological activity was investigated on iPSC-GABA after d 65. **(C)** *ZEB2* expression levels quantified by qRT-PCR during neurocortical differentiation of iPSC relative to expression of the housekeeping genes *ACTB* and *GAPDH*. MW-iPSC lines (*n* = 2), with a heterozygous c.1027C>T stop-variant (p.Arg343*) in exon 8, show reduced w.t. *ZEB2* mRNA levels (red line) when compared to that in Ctl-iPSC lines (*n* = 2; blue line) indicating haploinsufficiency. The total *ZEB2* mRNA levels in MW-iPSC lines are shown with a shaded red line. Data are presented as mean ± SEM (3 replicates per line). **(D)** Principal component analysis (PCA) of transcriptome data of iPSC, NSC and GABAergic cells derived from MWS patients (MW; *n* = 2) and healthy donors (Ctl; *n* = 2), respectively. The samples cluster according to differentiation time points and not according to *ZEB2* genotype.

The combined clinical findings made MWS the most likely diagnosis in both siblings. This was confirmed by targeted genetic investigation of the *ZEB2* gene that revealed a heterozygous nonsense variant c.1027C>T (p.Arg343*) in both siblings (Figure 1A). The variant, located in exon 8, was previously reported in 10 independent cases with MWS in the ClinVar (VCV000189281.15) database and it is absent from the normal population (gnomAD v2.1.1 database). The variant was therefore classified as Pathogenic (PVS1, PS4, PM2, PP4) according to the criteria from American College of Medical Genetics and Genomics (ACMG) (Richards et al., 2015). The variant was not detected in peripheral blood leukocytes of the healthy parents suggesting parental gonadal mosaicism.

### Induced pluripotent stem cells culture and neuronal differentiation

Induced pluripotent stem cell lines were previously established from fibroblasts of the two siblings (MW1, MW2) and two healthy donors (Ctl2, male and Ctl8, female, respectively) (Sobol et al., 2015; Schuster et al., 2019b). The four iPSC lines were matched for passage number (P25-P35), cultured in feeder free Essential-8*™* medium (ThermoFisher Scientific, Waltham, MA, United States) on either Matrigel*™*, Vitronectin*™* (Stem Cell Technologies, Vancouver, Canada) or LN521 (BioLamina) coated cell culture dishes and passaged as clumps with gentle cell dissociation reagent (GCDR; Stem Cell Technologies, Vancouver, Canada) or as single cells with TrypLExpress (ThermoFisher Scientific, Waltham, MA, United States) (Schuster et al., 2019b). Neurocortical differentiation of iPSCs was carried out as described using Dual-SMAD inhibition that promotes conversion into neural stem cells (NSC) for 10 days followed by directed differentiation into GABAergic cortical lineages for 55 days, i.e., totally 65 days (Figure 1B; Schuster et al., 2019a).

### Cell cycle and proliferation assay

The four iPSC lines were submitted to Dual Smad inhibition as described above and cultured to 70–80% confluence at d10 (i.e., iPSC-NSC). All samples were run in triplicates.

#### Proliferation assay

At day 10, iPSC-NSC were incubated with 10 μM EdU for 2 h and subsequently processed using a Click-it^®^ EdU Flow Cytometry Assay kit (ThermoFisher Scientific, Waltham, MA, United States) following recommended protocols. A total of 100,000 events was recorded for all samples on a Fortessa flow cytometer (BD Biosciences, NJ, United States) and subsequently analyzed using FlowJo 10.8.1. A sample of unlabeled cells was used to define the cut-off gate for EdU+ cells. The gate was applied to assess the number of actively dividing cells (i.e., EdU labeled cells) in labeled samples, where all events inside the gate were counted as EdU+.

#### Cell cycle analysis

Alternatively, iPSC-NSC at d10 were harvested with TryplExpress, fixed and resuspended in FxCycle™ PI/RNase staining solution (ThermoFisher Scientific, Waltham, MA, United States) to stain DNA. A total of 100,000 events was recorded as above and DNA content was plotted using FlowJo 10.8.1. Univariate cell cycle modeling was performed in FlowJo using the Dean-Jett-Fox model as described to derive percentages of cells in G1, S and G2 phase, respectively.

### Electrophysiological recordings

Whole-cell patch-clamp recordings were performed at room temperature (20–22°C) on differentiated GABAergic cells from day 65 showing a mature neuronal morphology, i.e., large and complex cell body with three or more neurites. Action potentials were evoked (eAPs) in response to step current injections in both Ctl-iPSC GABA and MW-iPSC GABA cells at a holding potential of −60 mV. An Axopatch 200B amplifier with the signal filtering filtered at 2 kHz, digitizing on-line at 10 kHz using an analog-to-digital converter and pClamp 10.2 software (Molecular Devices, USA) were used as described. Current steps were applied in 10 pA increments, each for 500 ms duration. Data were analyzed with pCLAMP software v10.5 and GraphPad PRISM (La Jolla, CA, USA).

### RNA isolation, quantitative real-time RT/PCR, and RNA sequencing

RNA from iPSC and neural cell populations was isolated using a miRNeasy micro kit (Qiagen, Hilden, Germany) and 1 μg of total RNA was reverse transcribed into cDNA using High Capacity cDNA transcription kit (ThermoFisher Scientific, Waltham, MA, United States). Expression of marker genes was compared to the two housekeeping genes *GAPDH* and *ACTB*. FastStart Universal SYBR Green Master mix (Roche, Basel, Switzerland) was used for qPCR with relevant primers. For all analyses, we used samples from three independent differentiation cultures and each sample was analyzed in triplicate. Expression Data (2^-dCT(vsAVERAGE (*GAPDH/ACTB*))) was plotted with the standard error of the mean (SEM) and fold change was presented as 2^-ddCT with SEM (*ACTB*: F-CAGGAGGAGCAATGATCTTGATCT, R-TCATGAAGTGTGACGTGGACATC; *GAPDH*: F-GAAGG TGAAGGTCGGAGTC, R-GAAGATGGTGATGGGATTTC; *ZEB2*: F-CGTTTCTCCCATTCTGGTTC, R-TGTGCGAACT GTAGGAACCA—located in exon 6 and exon 8, respectively, of *ZEB2* mRNA; *FOXG1*: F-CCCTCCCATTTCTGTACGTTT, R-CTGGCGGCTCTTAGAGAT; *VGLUT3*: F-GTCTAAGTGT GGGTCTCTTGTCA, R-TAGCCACCCCTTTGGTATGC; *BC AN*: F-CTCACGCTCGCGCAGTCT, R-TGTCTCCTTCCAG AACATCTGC; *LEF1*: F-AGAGCGAATGTCGTTGCTGA, R-G GCAGCTGTCATTCTTGGAC; *NEUROG2*: F-TGTTAGTGC TGCTCGGATCG, R-GTCTTCTTGATGCGCTGCAC; *NEUR OD6*: F-CAGGAGACGATGCGACACTC, R-TGCTTCTGGTC CTCGCATTC; *BARH2L*: For-AGACCAAACTCGACAAGC GG, R-ATTGAGCTGGTGGTCGGAAA; *EBF2*: F-CGGAGAT GGATTCGGTCAGG, R-TGAGTGCCGTTGTTGGTCTT; *EO MES*: F-GGGATCTTGCGGAGGACTGG, R-TGTAGTGGGC AGTGGGATTG; HMGA2: F-GGCAGCAAAAACAAGAGTC CC, R-ACTGCTGCTGAGGTAGAAATCG; *ITGB5*: F-GGAGA ACCAGAGCGTGTACC, R-AGCAGTTACAGTTGTCCCCG; *LRRK2*: F-CCTGTTGTGGAAGTGTGGGAT, R-TTCAGTATT TTTCCGGTTGTAGCC; *NEUROD1*: F-GAATTCGCCCACG CAGGA, R-ATCAGCCCACTCTCGCTGTA; *SEMA5A*: F-T CCACCTTCCCCGTGC, R-AGCCCAAGTCTCACACACCA; *POLR2A*: F-CACCGGCCTAGAGTTGTATGCGGAA, R-AAA CTTCCGCATACAACTCTAGGCC; *LHX6*: F-TCATAAAAAG CACACGCCGC, R-TATCGGCTTTGAGGTGGACG).

Paired-end RNA-sequencing libraries were prepared from 1 μg of total RNA using TruSeq stranded total RNA library preparation kit with RiboZero Gold treatment (Illumina, CA, United States) according to manufacturer’s protocols. Sequencing was performed on a HiSeq2500 (Illumina, CA, United States) with v4 sequencing chemistry on a total of 3 lanes at the SNP&SEQ Technology Platform, Science for Life Laboratory, Uppsala, Sweden.

### PCR, Sanger sequencing and quantification of *ZEB2* expression

We generated cDNA from iPSC and neural cell populations (see above) at specific time points of neurocortical differentiation for amplification of part of the *ZEB2* transcript containing the c.1027C>T variant with PCR primers (*ZEB2*: F-CGTTTCTCCCATTCTGGTTC, R-CCC GTGTGTAGCCATAAGAA). The PCR products were purified using a PCR clean up kit (MN) and Sanger sequenced (Eurofins Genomics, Ebersberg, Germany). The resulting DNA sequence chromatograms were analyzed using the QSVanalyzer software to estimate the relative abundance of wild type (w.t.) versus the variant allele (Carr et al., 2009).

### Immunofluorescent staining

Staining was performed on cells fixed with ice-cold 4% paraformaldehyde and subsequently permeabilized in blocking solution (1× phosphate-buffered saline pH 7.4, 1% bovine serum albumin, 0.1% Triton X-100). Primary antibodies against FOXG1 (1:100; abcam, Cambridge, United Kingdom), NESTIN (1:300; R&D systems, MN, United States), MAP2 (1:5,000; abcam, Cambridge, United Kingdom), GABA (1:1,000; Sigma, MO, United States), GAD1 (1:100, Millipore, MA, United States) and SST (1:100, Millipore, MA, United States) were used for immunostaining and quantification. Primary antibodies were allowed to bind overnight separately or in appropriate combinations at 4°C. After washing three times in 1×TBS, 0.05% Tween, the secondary antibodies donkey anti-goat IgG AlexaFluor 633, donkey anti-rabbit IgG AlexaFluor 568 or donkey anti-mouse IgG AlexaFluor 488 (1:1,000; ThermoFisher Scientific, Waltham, MA, United States) were applied alone or in appropriate combinations for 1.5 h at room temperature in the dark. Visualization was performed on a Zeiss 510 confocal microscope (Carl Zeiss Microscopy, Jena, Germany) using Zen 2009 imaging software.

### Wound healing scratch assay

At day 10 of neurocortical differentiation, MW-iPSC NCSs and Ctl-iPSC NSCs were re-seeded to obtain 100% confluence the following day. A pipette tip was used to scratch the culture introducing an artificial wound followed by bright field pictures taken at identical positions of the respective slide after 0, 15, and 20h. Closure of the open wound area was quantified using TScratch software (Geback et al., 2009). To circumvent data bias due to differences in the generation of wounds, the assay was repeated three times per iPSC cell line. Additionally, the TScratch analysis was performed as a time series experiment following closure of the wound area over time relative to the open area at start (open area at 20 h vs. open area at 0 h).

### Bioinformatic and statistical analysis

Bulk RNA sequencing reads were aligned to the ENSEMBL human reference genome (Homo_sapiens.GRCh37.75) and gene counts were generated using the STAR read aligner (Dobin et al., 2013). Number of expressed transcripts in each cell line was defined as all transcripts with more than one detected count (count > 1). Analysis of the count data to identify differentially expressed transcripts was performed using the DESeq2 package (design = ∼ condition) with 2 degrees of freedom (df = ncol(model.matrix(design(dds), colData(dds)))) (Love et al., 2014).

Gene ontology (GO) enrichment analysis using the PANTHER classification system^1^ was employed to analyze enrichment of DEGs in GO terms (Mi et al., 2013). Generation of networks was performed using Cytoscape and a STRING protein query with a confidence cut-off = 0.5 (Doncheva et al., 2019). Visualization and data presentation of gene enrichment was performed utilizing the cneplot software contained in the enrichplot R package^2^.

To evaluate our cell model of cortical GABAergic interneuronal development, we compared our data with single cell RNA-seq data from the developing human cortex (Nowakowski et al., 2017). Data was downloaded using UCSC cell browser^3^ and analyzed using Seurat 3 (Stuart et al., 2019). We performed an unbiased clustering using principal component analysis (PCA), followed by FindNeighbors (20 dimensions), FindClusters (resolution = 1.2), and UMAP dimensionality reduction visualization. We used subset data in Seurat 3 of clusters corresponding to interneurons, excitatory neurons and the neural precursors (Figure 4).

**FIGURE 2:**
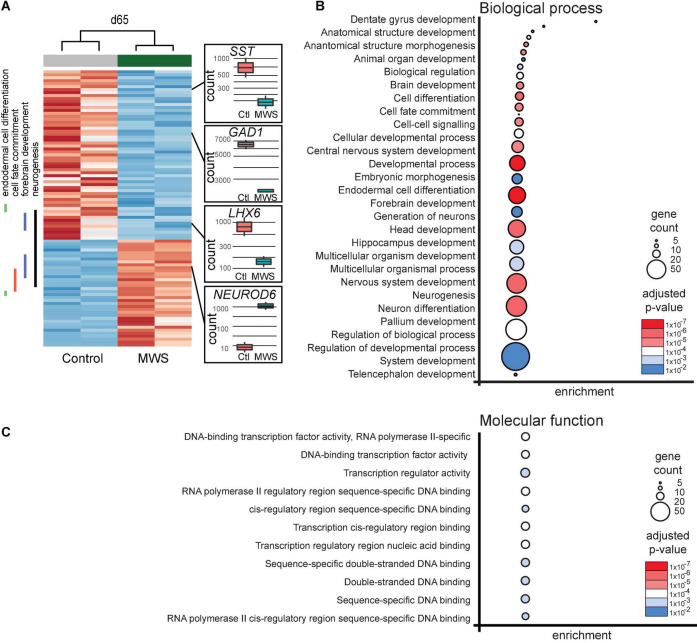
**(A)** Hierarchial clustering (z-scores) of 93 differentially expressed genes in two Ctl-iPSC GABA and two MW-iPSC GABA lines at d65 of differentiation from RNAseq. The expression (normalized counts) of selected genes (*SST*, *GAD1*, *LHX6*, *NEUROD6*) of importance for interneuronal development is highlighted as bar and whisker plots in Ctl-iPSC GABA (*n* = 2) and MW-iPSC GABA (*n* = 2). **(B,C)** GO enrichment analysis of 93 DEGs at d65 in MW-iPSC GABA cells showing the top 28 list of biological processes **(B)** and top 11 molecular functions **(C)**. Categories are listed according to adjusted p-values and number of DEGs in each category (gene count; Supplementary Tables 2A,C).

**FIGURE 3:**
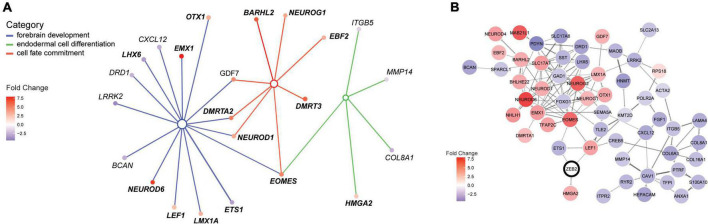
ZEB2 haploinsufficiency triggers differential expression of factors in a neural regulatory network. **(A)** Illustration of the interconnected network of DEGs belonging to enriched terms for the biological processes “Forebrain development” [GO:0030900; parent term to dentate gyrus development (GO:0021542); blue lines], “Endodermal cell differentiation” (GO:0035987; green lines) and “Cell fate commitment” (GO:0045165; red lines). The interconnected network was constructed using cnetplot. Interacting DEGs (filled circles) are colored according to gene expression (fold change [log2]). **(B)** STRING network with confidence edges illustrating documented protein interactions between ZEB2 (circled) and 55 out of 105 DEGs detected in our MW-iPSC derived neural cells at d10 and d65. DEGs (filled circles) are color-coded according to gene expression, as in panel **(A)**. The Network was generated using a STRING protein query [run in Cytoscape (Doncheva et al., 2019)] with a confidence cut-off = 0.5. ZEB2 is indicated with a bold outline.

**FIGURE 4:**
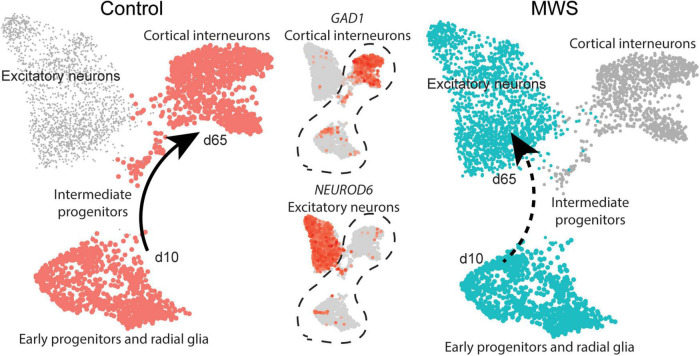
MW-iPSC with ZEB2 haploinsufficiency exhibit an aberrant interneuronal differentiation trajectory. The expression levels of *GAD1* and *NEUROD6* in Ctl-iPSC GABA (*n* = 2) and MW-iPSC GABA from RNAseq were inferred on pseudo-time trajectories of human cortex development. The expression of both genes in Ctl-iPSC GABA (left) are consistent with expression following the expected trajectory of cortical interneurons (Control, cell types highlighted in red). In MW-iPSC derived neural cells (MWS), the *GAD1* and *NEUROD6* expression suggests an early trajectory reminiscent of that for excitatory neuronal subtypes (dashed arrow).

Analysis of putative ZEB2 binding sites was performed using available Chip-Seq ENCODE data (ChIP-seq on eGFP-ZEB2 tagged human HEK293 cells: Accession ENCSR417VWF and for eGFP-ZEB2 tagged human K562 cells: Accession ENCSR322CFO). We lifted Chip-seq peaks (optimal IDR peaks i.e., high-confidence peaks for HEK293: Accession ENCFF232GGZ and for K562: Accession ENCFF129WGK) within 10 kb of our DEGs to identify putative ZEB2 binding sites.

## Results

### Differentiation of MW-iPSC and Ctl-iPSC generates GABAergic cells with gene expression profiles of forebrain

To get mechanistic insights into GABAergic development in MWS we used iPSC from the two siblings with a heterozygous and pathogenic *ZEB2* variant c.1027C>T (p.Arg343*; Figure 1A). Our differentiation protocol yielded a 90% enrichment of cells that stained positive for GABA and the key glutamate-to-GABA-synthesizing enzyme glutamate decarboxylase (GAD1) in both MW-GABA and Ctl-GABA cells (Supplementary Figure 1A; Schuster et al., 2019a). Approximately 30% of neuronal cells stained positive for somatostatin (SST), a marker for a large subpopulation of inhibitory interneurons, in all lines (Lim and Marin, 2018). There were no significant differences in growth, morphology or staining of the GABAergic markers when comparing MW-iPSC and Ctl-iPSC derived neural lines (Supplementary Figures 1A,B).

The *ZEB2* variant predicts a truncated protein lacking essential functional domains such as the Smad-binding domain, the Homeodomain-like domain, the consensus interaction sequences for CtBP-1/2 co-repressors and the C-terminal zink-finger cluster (Figure 1A; Epifanova et al., 2018; Birkhoff et al., 2021). The iPSC derived from the two MWS subjects (MW1-iPSC and MW2-iPSC) were induced for neural differentiation together with iPSC from two healthy donors (Ctl2-iPSC and Ctl8-iPSC) during 10 d to obtain neural stem cells (NSCs) followed by differentiation into GABAergic forebrain lineages for additional 55 days (Figure 1B; Chambers et al., 2009; Schuster et al., 2019a). To investigate ZEB2 haploinsufficiency in the MW-iPSCs we analyzed *ZEB2* mRNA levels at different time-points along neural induction and neurocortical differentiation until day 65 (Figure 1B). The total *ZEB2* expression increased upon neural induction in all lines from day 0–3 and remained stable from day 10 until day 65 at similar levels in both MW-iPSC and Ctl-iPSC. However, sequence analysis of *ZEB2* RNA across the c.1027C>T variant revealed that the variant was present in approximately 50% of transcripts in the MWS subject derived iPSC lines at all time points (Figure 1C). Consequently, the w.t. *ZEB2* levels in the two MW-iPSC lines were approximately halved when compared to Ctl-iPSC throughout differentiation supporting haploinsufficiency for w.t. ZEB2 (Figure 1C).

We then performed RNA-sequencing (RNAseq) and assessed the gene expression profiles of MW-iPSC and Ctl-iPSC at day 0 (undifferentiated iPSCs), in NSCs at d10 (iPSC-NSC), and after differentiation into postmitotic GABAergic interneurons (iPSC-GABA) at day 65 (Figure 1B). We obtained on average 34.7 million reads (ranging from 24.4 × 10^6^ to 42.7 × 10^6^ reads) from the four lines at all time points. The average numbers of expressed transcripts (>1 count) were 26,180 at day 10 and 28,140 at day 65, respectively. Normalized data were used to calculate the Euclidean distance between samples and all samples clustered according to differentiation time points (Supplementary Figure 1C). In addition, principal component analysis (PCA) confirmed that the overall differences in transcriptomes were related to the differentiation time-points and not to the *ZEB2* genotype suggesting similar overall cell compositions in MW-iPSC and Ctl-iPSC (Figure 1D; Schuster et al., 2019a). Furthermore, analysis at the three time-points revealed changes in expression (log2(counts)) of neural genes related to the differentiation for each iPSC line (Supplementary Figure 2A). The transcriptomic profiles of each of the four lines at day 65 were further validated by comparing the 5,000 most highly expressed transcripts with expression profiles across different brain regions obtained from the Human Gene Atlas and the Allen Brain Atlas (Sunkin et al., 2013; Miller et al., 2014). All four lines showed the closest similarities with transcriptomes of “Prefrontal Cortex” and “Fetal Brain” (Human Gene Atlas; EnrichR combined score > 50), “Dentate gyrus” and “Superficial dorsofrontal area” (Allen Brain Atlas; EnrichR combined score > 50) in neonates (Schuster et al., 2019a).

### Differentially expressed genes in MW-iPSC derived GABAergic neurons are enriched in gene ontology terms for cell fate, forebrain development and transcriptional regulation

We next performed a GO analysis using differentially expressed genes (DEGs) in the MW-iPSC vs. Ctl-iPSC lines. The comparison of RNAseq data between the non-induced MW-iPSC and Ctl-iPSC lines revealed similar patterns. However, in NSC we identified 12 DEGs (adjusted *p*-value < 0.05; Supplementary Table 1A) and in GABAergic interneurons the number of DEGs was 93 (adjusted *p*-value < 0.05; Supplementary Table 1B; Figure 2A). The RNAseq data was validated by qRT-PCR analysis of 16 DEGs confirming up-regulation (*n* = 8) or downregulation (*n* = 8), respectively (Supplementary Figures 2B,C). Expression of two interneuronal markers (*SST* and *GAD1)* at day 65 revealed down-regulation in MW-GABA compared to Ctl-GABA cells from our RNAseq analysis (Log2fold changes −1,5 and −1,1, respectively; Supplementary Tables 1B,C). However, the qRT-PCR analysis of both genes did not reach significant differences between the sample groups.

To identify enriched GO terms for biological processes we then analyzed the DEGs in a GO enrichment analysis. The analysis revealed no significant enrichment for DEGs in NSCs at d10. However, when applying the 93 DEGs at d65 we identified the top specific term “Dentate gyrus development” [GO:0021542; 5 DEGs (5%); Figure 2B; Supplementary Tables 2A,B] among the most enriched categories. The term is placed in a hierarchy under “Forebrain development” [GO:0030900; 20 DEGs (21%)]. Among the top terms we also identified “Cell fate commitment” [GO:0045165; 14 DEGs (15%)] and “Endodermal cell differentiation” [GO:0035987; 24 DEGs (26%); Figure 2B; Supplementary Tables 2A,B]. Notably, nine DEGs were represented in more than one of the enriched terms for biological processes and four of these DEGs (*GDF7*, *DMRTA2, NEUROD1*, and *EOMES*) formed an interconnected network comprising 23 DEGs (Figure 3A). We observed that 15 out of the 23 DEGs are TFs (Table 1) and we therefore analyzed the set of 93 DEGs d65 for enrichment in GO terms for molecular function. The analysis revealed the top terms “DNA-binding transcription factor activity, RNA polymerase II-specific” (GO:0000981; 22 DEGs), placed in a hierarchy under the term “Transcription regulator activity” (GO:0140110; 25 DEGs), and the similar term “RNA polymerase II regulatory region sequence-specific DNA binding” (GO:0000977; 23 DEGs; Supplementary Tables 2C,D; Figure 2C). The entire set of DEGs at day 10 and 65 (n = 105) comprised altogether 28 TFs and transcriptional regulators (27%; Supplementary Tables 1A,B).

**TABLE 1:** Differentially expressed genes (DEGs) identified in MW-iPSC NSC and MW-iPSC GABA that belong to the top enriched GO terms for biological processes and molecular functions.

Gene symbol	Gene name	log2 Fold change^a^	p-adj^b^	GO term			
				FD^c^	CFC^c^	ECD^c^	TF^d^
**iPSC-NSC (d10)**							
FOXG1	Forkhead box protein G1	–1.2	0.0099178	X	X		X
SEMA5A	Semaphorin-5A	–1.2	0.0045	X			
**iPSC-GABA (d65)**							
BARHL2	BarH-like 2 homeobox protein	1.7	0.0085956		X		X
BCAN	Brevican core protein	–1.8	0.0007659	X			
COL8A1	Collagen alpha-1(VIII) chain	–1.7	0.0042997			X	
CXCL12	Stromal cell-derived factor 1	–1.7	0.0033663	X			
DMRT3	Doublesex- and mab-3-related transcription factor 3	2.3	1.58*E*−05		X		X
DMRTA2	Doublesex- and mab-3-related transcription factor A2	2.3	2.23*E*−05	X	X		X
DRD1	D(1A) dopamine receptor	–1.9	0.0017525	X			
EBF2	Transcription factor COE2	1.5	0.0397296		X		X
EMX1	Homeobox protein EMX1	2.0	0.0004469	X			X
EOMES	Eomesodermin homolog	3.2	7.41*E*−14	X	X	X	X
ETS1	Protein C-ets-1	–1.5	0.0397296	X			X
GDF7	Growth/differentiation factor 7	1.6	0.020917	X	X		
HMGA2	High mobility group protein HMGI-C	1.7	0.0008172			X	X
ITGB5	Integrin beta-5	–1.2	0.0247298			X	
LEF1	Lymphoid enhancer-binding factor 1	1.9	1.15*E*−05	X			X
LHX6	LIM/homeobox protein Lhx6	–1.8	0.0010726	X			X
LMX1A	LIM homeobox transcription factor 1-alpha	1.5	0.0452728	X			X
LRRK2	Leucine-rich repeat serine/threonine-protein kinase 2	–1.6	0.0284824	X			
MMP14	Matrix metalloproteinase-14	–1.1	0.0409523			X	
NEUROD1	Neurogenic differentiation factor 1	–1.1	0.0409523	X	X		X
NEUROD6	Neurogenic differentiation factor 6	4.0	1.06*E*−21	X			X
NEUROG1	Neurogenin-1	1.5	0.0385899		X		X
OTX1	Homeobox protein OTX1	1.8	0.0035067	X			X

^*a*^relative to expression in Ctl-iPSC GABA.

^*b*^adjusted p-value after Bonferroni correction.

^*c*^X indicates gene is contained in the GO terms biological process: FD, forebrain developent; CFC, cell fate committment; ECD, endodermal cell differentiation.

^*d*^X indicates gene is contained in the GO terms molecular function: TF, transcription regulator activity.

To further validate interactions among the 93 DEGS at day 65, we then used the Search Tool for the Retrieval of Interacting Genes (STRING). The analysis revealed a protein-protein network that comprised 55 proteins encoded by the DEGs (Figure 3B). Moreover, we sought to get mechanistic insights into a possible role of ZEB2 on the regulation of individual DEGs among the top significantly enriched terms. We therefore searched for ZEB2 binding sites among our DEGs using available CHIPseq data (ENCODE) derived from HEK293 and K562 cells. Among the 12 DEGs in NSCs, ZEB2 binding was reported in 10 (83%; Supplementary Table 1A; see examples in Supplementary Figure 2D) and in 65 among the 93 DEGs in GABAergic cells (70%; Supplementary Table 1B). This observation suggests a direct involvement of ZEB2 in the regulation of a large proportion of DEGs identified in our model system.

Taken together, analysis of the gene expression changes in MW-iPSC GABA interneurons revealed enrichment of genes in an interconnected gene regulatory network for biological processes such as cell fate decision, neural patterning and specification.

### Transcriptional changes in ZEB2 haploinsufficient GABAergic cells uncover a disrupted identity and dysregulations linked to epilepsy genes

Beyond the DEGs belonging to the top enriched terms, we identified several additional and dysregulated genes important for interneuronal development when comparing MW-iPSC with Ctl-iPSC derived NSCs and GABAergic neurons, respectively. For example, MW-subject derived NSCs showed downregulation of the transcriptional regulators *KMT2D* and *FOXG1*, essential for neuronal lineage commitment (Wang et al., 2016) and neocortical organization (Cargnin et al., 2018). Furthermore, we observed upregulation of the genes encoding the TFs NEUROG2, NEUROD4 and NHLH1 (NSCL1) that promote glutamatergic development while suppressing genes for microglia identity (Roybon et al., 2010; Kim, 2012; Guillemot and Hassan, 2017; Dennis et al., 2019; Aslanpour et al., 2020; Liu et al., 2021; Tutukova et al., 2021). The combined transcriptional changes predict a disrupted GABAergic identity in MW-iPSC and we therefore sought to simulate the lineage trajectory of GABAergic development in our models. To this end we selected two DEGs d65 from our RNAseq data encoding the down-regulated GABAergic marker GAD1 and the upregulated excitatory marker NEUROD6 (Figure 2A and Supplementary Table 1). We then inferred the expression levels of the two DEGs on pseudo-time trajectories based on gene expression data available from single cells along human cortical development (Nowakowski et al., 2017). In Ctl-iPSC GABA cells the expression levels of both genes displayed trajectories in agreement with that of cortical interneurons originating from early neural progenitors and radial glia, confirming that our protocol generates cortical GABAergic interneurons. In contrast, the expression levels of *NEUROD6* and *GAD1* in MW-iPSC GABA cells simulated a differentiation trajectory directed toward excitatory neurons (Figure 4 and Supplementary Table 1B).

Moreover, given the association between altered GABAergic function and seizures, we then sought to investigate possible interactions of all DEGs with genes associated with epilepsy. We therefore compiled a list of 208 unique genes associated with genetic epilepsy, neurodevelopment and epilepsy syndromes from two recent studies (Supplementary Table 3; Wang J. et al., 2017; Jang et al., 2019). Subsequently, we re-analyzed the 105 DEGs together with ZEB2 and the 208 curated epilepsy-associated genes for possible protein-protein interactions using STRING (in total 314 distinct genes; Supplementary Table 3; Wang J. et al., 2017; Jang et al., 2019). The analysis disclosed a multiplex network that extended our protein-protein network with 282 of the queried 314 epilepsy-associated genes (89.8%; Supplementary Figure 3; Supplementary Table 3).

### Neural progenitors derived from MWS-patient iPSC exhibit migration defects

**FIGURE 5:**
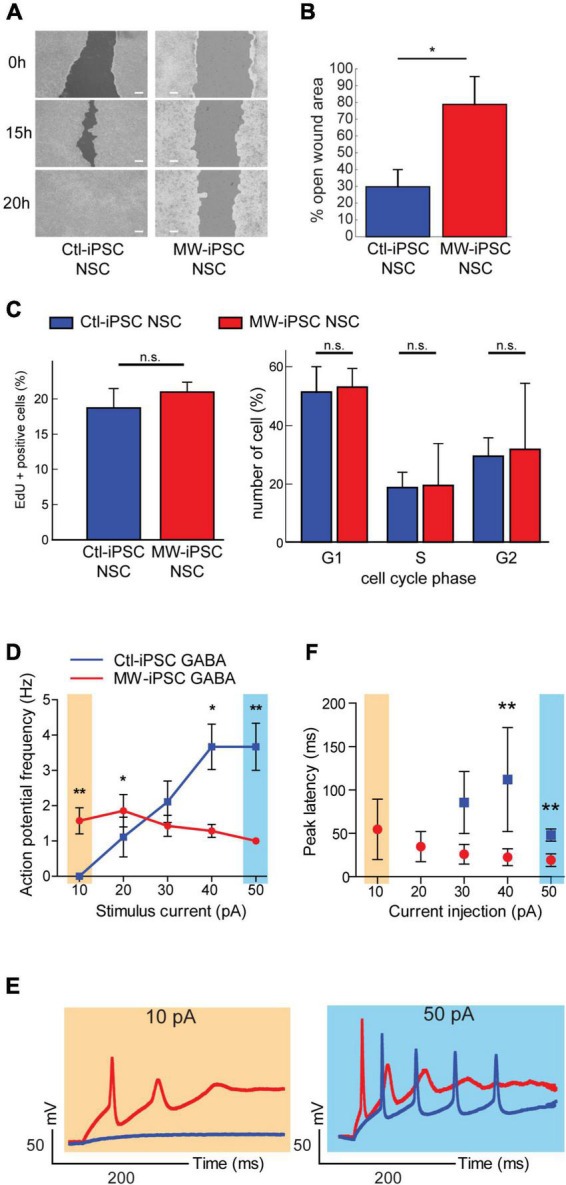
MW-iPSC derived neural cells with ZEB2 haploinsufficiency show impaired migration and altered electrophysiology. **(A,B)** Assessment of cell motility of iPSC-NSCs at day 10 using a wound healing scratch assay shows impaired motility in MW-iPSC NSCs. **(A)** Confluent cultures of MW-iPSC NSCs and Ctl-iPSC NSCs at day 10 were scratched and imaged at identical positions after 0 h, 15 h and 20 h. Representative pictures are shown. Size bar 200 μm. **(B)** Quantification of open wound area in MW-iPSC NSCs and Ctl-iPSC NSCs using TScratch software. The remaining open area was quantified at 15 h vs. 0 h (3 replicates per iPSC line) and significantly larger in MW-iPSC NSCs when compared to Ctl-iPSC NSCs (*p < 0.05). **(C)** Assessment of Proliferation of iPSC-NSC at day 10 using EdU proliferation assay and cell cycle analysis show similar proliferation activity of patient and control iPSC derived cells (n.s., not significant; *n* = 4 replicates per cell line). **(D–F)** MW-iPSC GABA cells show aberrant firing of action potentials and shorter peak latency time in response to step current injections. **(D)** The maximal frequency of evoked action potentials (eAPs) was markedly reduced in MW-iPSC GABA cells (red line) as compared to control cells (blue line; eAPs (Hz) plotted versus the stimulus current (pA) used). **(E)** Responses to 10 pA (yellow) (left panel) and 50 pA (blue) (right panel) current injections lasting 500 ms in representative MW-iPSC GABA and Ctl-iPSC GABA cells at a holding potential of –60 mV. At a depolarizing current stimulus of 10 pA the MW-iPSC GABA cells were able to fire single eAPs while Ctl-iPSC GABA cells remained silent. At a depolarizing current stimulus of 50 pA, MW-iPSC GABA cells fired still only single eAPs while Ctl-iPSC GABA cells fired multiple consecutive eAPs. **(F)** The peak latency (delay time; ms) of iPSC-GABA cells in response to the injected stimulus (pA) was significantly reduced in MW-iPSC GABA cells at current injections >30 pA. **p < 0.005, *p < 0.05.

In mice, the conditional loss of Zeb2 causes failure of interneuronal precursor cells to migrate tangentially into the cortex (McKinsey et al., 2013; van den Berghe et al., 2013; He et al., 2018). These prior reports, together with the observed downregulation of *LHX6*, a marker for interneuronal migration and laminar positioning (Liodis et al., 2007), in our ZEB2 haploinsufficient GABAergic cells prompted us to investigate the migratory capacity of our iPSC derived NSCs. To test this, we established confluent MW-iPSCs and Ctl-iPSCs derived NSC cultures and performed a wound healing scratch assay for 20 h. Analysis of both Ctl-iPSC NSC cultures revealed that the open area became almost closed after 15 h and completely closed after 20 h post scratching. In contrast, the two MW-iPSC NSC cultures were only partially closed after 20 h (Figures 5A,B; *p*-value = 0.031). To exclude a possible effect of different proliferative capacity on the results we then performed a proliferation assay on MW-iPSC NSC and Ctl-iPSC NSCs. There were no detectable differences in proliferative capacity between the two groups (Figure 5C). These findings suggest an impaired migratory capacity in MW-iPSC NSCs.

### MW-iPSC derived GABAergic interneurons show impaired firing of action potentials

Given the preponderance of seizures in MWS together with the distorted molecular identity of our ZEB2 haploinsufficient GABAergic cells, we sought to investigate if our model was associated with changes in electrophysiological properties. We therefore assessed the electrophysiology of the ZEB2 haploinsufficient GABAergic cells using whole-cell patch clamp analysis. Upon stimulation, the Ctl-iPSC GABA cells required a stimulus of 20 pA or more to fire evoked action potentials (eAPs) whereas the MW-iPSC GABA cells were able to fire one or rarely two eAP at a depolarizing current stimulus of 10 pA (Figures 5D–E). Furthermore, the majority of MW-iPSC GABA cells discharged only one single eAP during the depolarizing current injection, irrespective of the injected activation stimulus. In contrast, the Ctl-iPSC GABA cells showed repetitive eAP spikes that increased in number with the strength of current injection until reaching a maximum number of spikes (Figures 5D,E). In Ctl-iPSC GABA cells the maximal number of eAPs was 6 at a stimulus of 50 pA with an average amplitude of APs of 84 ± 10 mV, comparable to that of the single eAP in MW-iPSCs (82 ± 11 mV; Figure 5F). In addition, while the first eAP amplitude in MW-iPSC and Ctl-iPSC GABA cells showed no significant differences, the delay time for the cells to react to the injected stimulus (i.e., peak latency) was significantly reduced in MW-iPSC GABA cells (Figure 5F). Altogether, the electrophysiological analysis shows that MW-iPSC GABA cells respond to lower stimuli when compared to Ctl-iPSC GABA cells but fail to generate subsequent APs, usually required for signal transfer and intercellular activation. The electrophysiological analysis thus indicates impaired biophysical properties of ZEB2 haploinsufficient MW-iPSC GABA cells.

## Discussion

The present study was motivated by questions on specific abnormalities in elements, pathways and functions for the development of MWS-subject derived GABAergic cells, a key cell type for the regulation of microcircuitries and ictal activity, to gain insights into mechanisms behind the neuropathology in MWS. We therefore generated NPCs and GABAergic cells derived from iPSCs of two MWS subjects with haploinsufficiency for the w.t. ZEB2 protein and from two healthy donors. Following neural patterning of iPSCs, the differentiation protocol yielded GABAergic interneurons with 90% efficiency in all four lines of which 30% expressed the interneuronal marker SST. The expression of markers NPY, PV, and VIP (Lim et al., 2018) were very low to neglectable, suggesting similarities of our model to that of interneuronal development from hESC (Close et al., 2017). Moreover, our GABAergic cells recapitulated transcriptional programs of neonatal brain regions that coincide with structural changes observed in cases with MWS (Garavelli et al., 2017). Together, this suggest that our model system provides a context to study molecular and functional perturbations of GABAergic interneurons relevant for MWS.

We focused our molecular analysis on protein coding sequences obtained from deep RNAseq at two time-points of GABAergic differentiation, thereby increasing the possibilities to capture temporal gene expression changes associated with ZEB2 haploinsufficiency. Because of the limited number of biological replicates available (df = 2) in our study we did not expect to detect subtle gene expression differences between the two groups. We therefore used DESeq2 to identify the most pronounced changes in MWS subject derived iPSC (Schurch et al., 2016). The analysis highlights a set of co-dysregulated genes in MW-iPSC GABA cells that are markedly enriched in transcriptional regulation and in interconnected biological processes. The gene expression pattern in the ZEB2 haploinsufficient neural model revealed an attenuated and immature identity of ZEB2 haploinsufficient GABAergic cells, with features of excitatory, glutamatergic and midbrain neuronal cells. For example, in MW-iPSC NSC we observed downregulation of the pan-telencephalic marker *FOXG1* acting as a suppressor of *NEUROG2* expression (Yang et al., 2017; Cargnin et al., 2018). Consequently, we observed increased expression of *NEUROG2*, as well as of *NEUROG1* that inhibits neocorticogenesis, and their downstream targets *NEUROD1*, *NEUROD4* and *NEUROD6* (Schuurmans et al., 2004; Tutukova et al., 2021). The TF NEUROG2 suppresses the microglia program and compromises GABAergic subtype specification (Roybon et al., 2010; Matsuda et al., 2019) while promoting the differentiation of cortical deep layer glutamatergic neurons together with NEUROD1, NEUROD4, and NEUROD6 (Gascon et al., 2017; Han et al., 2018). Since *FOXG1* was identified as a ZEB2 target gene from available ChiP seq data, our ZEB2 haploinsufficient model thus suggests a dysregulation of the transcriptional axis for GABAergic development involving the TFs ZEB2-FOXG1-NEUROG-NEUROD. In addition, MW-GABA cells showed a marked increase in expression of the TF genes *EMX1*, *EOMES/TPR2*, *LMX1* and *LEF1* promoting organization of excitatory progenitor cells in the pallium (Kobeissy et al., 2016; Armenteros et al., 2018; Hevner, 2019; Lv et al., 2019; Nouri et al., 2020). In the mouse model depleted of Zeb2 in the medial ganglionic eminence (MGE) from E9, the Zeb2 induced repression of Nkx2-1 is lost leading to cortical interneurons transformed toward a striatal interneuronal subtype characterized by TACR1, NPY, nNOS, and SST expression (McKinsey et al., 2013). Our RNA-seq data from ZEB2 haploinsufficient GABAergic cells d65 showed only a tendency for downregulation of *NKX2-1* together with the striatal markers *TACR1, NPY, NRP1*, and *NOS1*. The reason for the different expression pattern of some striatal markers in our system when compared to the mouse model is unclear but a possible explanation for the incomplete repression of NKX2-1 is the remaining expression of *ZEB2* from the w.t. allele. Another contributing reason could be the differentiation protocol and the 2D culture system used in our study that strongly promotes neocortical lineages.

In mice, the conditional depletion of neural Zeb2 results in a partially stalled migration of cortical Lhx6 positive interneurons (McKinsey et al., 2013) and a misrouting into the ventral and caudal part of telencephalon (van den Berghe et al., 2013). The DNA binding protein LHX6 promotes SST expression, tangential migration, specification and laminar positioning of GABAergic interneurons (Liodis et al., 2007; Miyoshi and Fishell, 2011; Kessaris et al., 2014; Kim et al., 2021). In line with neural Zeb2 deficiency in mice, we observed a reduced expression of *LHX6* in MW-iPSC GABA cells and a marked migration deficiency in MW-iPSC NSCs uncovered by our wound healing assay. These findings support a perturbed GABAergic development and a disrupted program for interneuronal migration in our ZEB2 haploinsufficient model that are relevant for the developmental brain abnormalities observed in MWS subjects.

GABAergic interneurons are critical to maintain the balance between neuronal excitation and inhibition for proper cortical functions. Consistent with this role, malfunction of inhibitory interneurons has been associated with different types of epilepsy. Our multilayer STRING analysis of known epilepsy associated genes together with the 105 DEGs identified in our study uncovered extensive connections, suggesting downstream effects of ZEB2 haploinsufficient GABAergic development on pathways and factors interfering with ictal activity. This is supported by the electrophysiological properties in our MWS model of GABAergic interneurons. Action potentials were considerably fewer and did not respond to increased current injections in MW-iPSC GABA when compared to Ctl-iPSC GABA cells, possibly because of a concomitant reduced peak latency and impaired ability to re-polarize in response to stimuli. Our observations from whole cell patch-clamp analysis of ZEB2 haploinsufficient GABAergic inhibitory interneurons thus indicate a hypoexcitable cell phenotype with impaired responses to stimuli that may contribute to seizures observed in the majority of cases with MWS.

Among its versatile functions, ZEB2 plays a critical role for neuroectoderm formation, ectodermal-to-mesenchymal transition (EMT) and crest cell induction (Epifanova et al., 2018). Accordingly, our ZEB2 haploinsufficient model exhibited altered expression of several factors regulating EMT. For example, upregulation was observed for the TF TFAP2C that maintains pluripotency and promotes mesoderm-to-epithelial transition (MET) by repressing neuroectodermal differentiation (Pastor et al., 2018) as well as of the gene encoding KLHL14, that inhibits EMT (Di Lollo et al., 2020), and HMGA2, promoting EMT (Thuault et al., 2008; Kou et al., 2018). This was accompanied by a downregulation of the ZEB2 target gene ETS1 (Theveneau et al., 2007; Wang et al., 2015; Yalim-Camci et al., 2019), maintaining EMT, together with genes for the extracellular matrix proteins MMP14, ITGB5 and COL8A1 that promote delamination of neural crest cells and EMT induction. These data bring further support for a disturbed cell fate switch during GABAergic development that complies with prior data showing dysregulation of EMT and crest cell induction after ZEB2 downregulation (Van de Putte et al., 2007; Chng et al., 2010), as well as the crest cell derived features associated with MWS (Ivanovski et al., 2018).

Our study of ZEB2 haploinsuffient neural cells focused on GABAergic development because of the key regulatory roles on neural circuitries and for preventing harmful ictal activity, a feature observed in the majority of MWS cases. However, ZEB2 is widely expressed in the developing central and peripheral nervous system. Prior studies have shown *Zeb2* expression in, e.g., oligodendrocytes, Bergmann glia cells and brainstem neurons (Nishizaki et al., 2014). Therefore, a perturbed development of a range of brain cell types are likely in MWS in addition to the effects of ZEB2 haploinsufficiency shown and predicted in our study. More complex models of neural differentiation with ZEB2 haploinsufficiency using, e.g., 3D organoids, build up by a mixture of cell types, followed by single cell transcriptome analysis may add important findings to our study.

Taken together, analysis of our ZEB2 haploinsufficient iPSC model of GABAergic development uncover transcriptional changes consistent with a disrupted cell fate switch and an immature and attenuated identity of inhibitory interneurons. This is associated with impaired migratory capacity in neural progenitor cells and hypoexcitable GABAergic interneurons. The combined findings thus suggest perturbed development and function of GABAergic cells in MWS with implications for the formation of different brain structures and for balancing neuronal circuitries.

## Conclusion

We show that an iPSC model of GABAergic development in a ZEB2 haploinsufficient iPSC model of MWS exhibits dysregulations of specific genes along interneuronal differentiation. The dysregulated genes form an interconnected network enriched for transcriptional regulation, cell fate decision and forebrain formation, with extensive connections to genes implicated in epilepsy. Furthermore, our MWS model of GABAergic development exhibits a gene expression profile mixed with that of midbrain and glutamatergic development, leading to an immature and attenuated GABAergic identity. The transcriptional changes in ZEB2 haploinsufficient neural cells are associated with impaired migration of NSCs and altered electrophysiological properties of GABAergic interneurons that correlate with clinical features observed in MWS. The comprehensive data from our study provide a framework for further studies of neuronal development in MWS with the long-term goal to interfere with brain development and seizures in affected individuals.

## Data availability statement

The data presented in this study are deposited in the SciLifeLab data repository, accession number 20472144 (10.17044/scilifelab.20472144.v1).

## Ethics statement

Written informed consent was obtained from the minor(s)’ legal guardian/next of kin for the publication of any potentially identifiable images or data included in this article.

## Author contributions

JS: conceptualization, methodology, formal analysis, investigation, writing—original draft and review and editing, visualization, and project administration. JK: methodology, software, data curation, writing—review and editing, and visualization. AK, LL, ZJ, and SVK: methodology, formal analysis, and investigation. JH: methodology, validation, and investigation. AF and VS: methodology and investigation. MH: software, validation, data curation, and visualization. AN and BMA: resources. CG: validation, formal analysis, and funding acquisition. BB: software, formal analysis, supervision, and funding acquisition. ND: conceptualization, formal analysis, and writing—review and editing, visualization, supervision, project administration, and funding acquisition. All authors contributed to the article and approved the submitted version.

## Footnotes

1
geneontology.org

2
https://www.bioconductor.org/packages/release/bioc/html/enrichplot.html

3
cells.ucsc.edu

## References

[B1] Adam M. P. Schelley S. Gallagher R. Brady A. N. Barr K. Blumberg B. (2006). Clinical features and management issues in Mowat-Wilson syndrome. *Am. J. Med. Genet. A* 140 2730–2741. 10.1002/ajmg.a.31530 17103451

[B2] Armenteros T. Andreu Z. Hortiguela R. Lie D. C. Mira H. (2018). BMP and WNT signalling cooperate through LEF1 in the neuronal specification of adult hippocampal neural stem and progenitor cells. *Sci. Rep.* 8:9241. 10.1038/s41598-018-27581-0 29915186 PMC6006330

[B3] Aslanpour S. Han S. Schuurmans C. Kurrasch D. M. (2020). Neurog2 acts as a classical proneural gene in the ventromedial hypothalamus and is required for the early phase of neurogenesis. *J. Neurosci.* 40 3549–3563. 10.1523/JNEUROSCI.2610-19.2020 32273485 PMC7189762

[B4] Birkhoff J. C. Huylebroeck D. Conidi A. (2021). ZEB2, the Mowat-Wilson syndrome transcription factor: Confirmations, novel functions, and continuing surprises. *Genes (Basel)* 12:1037. 10.3390/genes12071037 34356053 PMC8304685

[B5] Brinkmann B. G. Quintes S. (2017). Zeb2: Inhibiting the inhibitors in Schwann cells. *Neurogenesis (Austin)* 4:e1271495. 10.1080/23262133.2016.1271495 28203609 PMC5293318

[B6] Cargnin F. Kwon J. S. Katzman S. Chen B. Lee J. W. Lee S. K. (2018). FOXG1 orchestrates neocortical organization and cortico-cortical connections. *Neuron* 100 1083.e–1096.e. 10.1016/j.neuron.2018.10.016 30392794 PMC6428593

[B7] Carr I. M. Robinson J. I. Dimitriou R. Markham A. F. Morgan A. W. Bonthron D. T. (2009). Inferring relative proportions of DNA variants from sequencing electropherograms. *Bioinformatics* 25 3244–3250. 10.1093/bioinformatics/btp583 19819885

[B8] Chambers S. M. Fasano C. A. Papapetrou E. P. Tomishima M. Sadelain M. Studer L. (2009). Highly efficient neural conversion of human ES and iPS cells by dual inhibition of SMAD signaling. *Nat. Biotechnol.* 27 275–280. 10.1038/nbt.1529 19252484 PMC2756723

[B9] Chng Z. Teo A. Pedersen R. A. Vallier L. (2010). SIP1 mediates cell-fate decisions between neuroectoderm and mesendoderm in human pluripotent stem cells. *Cell Stem Cell* 6 59–70. 10.1016/j.stem.2009.11.015 20074535

[B10] Close J. L. Yao Z. Levi B. P. Miller J. A. Bakken T. E. Menon V. (2017). Single-cell profiling of an *in vitro* model of human interneuron development reveals temporal dynamics of cell type production and maturation. *Neuron* 93 1035.e–1048.e. 10.1016/j.neuron.2017.02.014 28279351 PMC5480972

[B11] Dennis D. J. Han S. Schuurmans C. (2019). bHLH transcription factors in neural development, disease, and reprogramming. *Brain Res.* 1705 48–65. 10.1016/j.brainres.2018.03.013 29544733

[B12] Di Lollo V. Canciello A. Orsini M. Bernabo N. Ancora M. Di Federico M. (2020). Transcriptomic and computational analysis identified LPA metabolism, KLHL14 and KCNE3 as novel regulators of epithelial-mesenchymal transition. *Sci. Rep.* 10:4180. 10.1038/s41598-020-61017-y 32144311 PMC7060278

[B13] Dobin A. Davis C. A. Schlesinger F. Drenkow J. Zaleski C. Jha S. (2013). STAR: Ultrafast universal RNA-seq aligner. *Bioinformatics* 29 15–21. 10.1093/bioinformatics/bts635 23104886 PMC3530905

[B14] Doncheva N. T. Morris J. H. Gorodkin J. Jensen L. J. (2019). Cytoscape stringapp: Network analysis and visualization of proteomics data. *J. Proteome Res.* 18 623–632. 10.1021/acs.jproteome.8b00702 30450911 PMC6800166

[B15] Epifanova E. Babaev A. Newman A. G. Tarabykin V. (2018). Role of Zeb2/Sip1 in neuronal development. *Brain Res.* 1705 24–31. 10.1016/j.brainres.2018.09.034 30266271

[B16] Galanopoulou A. S. (2010). Mutations affecting GABAergic signaling in seizures and epilepsy. *Pflugers Arch.* 460 505–523. 10.1007/s00424-010-0816-2 20352446 PMC2885462

[B17] Garavelli L. Mainardi P. C. (2007). Mowat-Wilson syndrome. *Orphanet J. Rare Dis.* 2:42. 10.1186/1750-1172-2-42 17958891 PMC2174447

[B18] Garavelli L. Ivanovski I. Caraffi S. G. Santodirocco D. Pollazzon M. Cordelli D. M. (2017). Neuroimaging findings in Mowat-Wilson syndrome: A study of 54 patients. *Genet. Med.* 19 691–700. 10.1038/gim.2016.176 27831545 PMC5438871

[B19] Gascon S. Ortega F. Gotz M. (2017). Transient CREB-mediated transcription is key in direct neuronal reprogramming. *Neurogenesis (Austin)* 4:e1285383. 10.1080/23262133.2017.1285383 28321434 PMC5345748

[B20] Geback T. Schulz M. M. Koumoutsakos P. Detmar M. (2009). TScratch: A novel and simple software tool for automated analysis of monolayer wound healing assays. *Biotechniques* 46 265–274. 10.2144/000113083 19450233

[B21] Gouwens N. W. Sorensen S. A. Baftizadeh F. Budzillo A. Lee B. R. Jarsky T. (2020). Integrated morphoelectric and transcriptomic classification of cortical GABAergic cells. *Cell* 183 935.e–953.e. 10.1016/j.cell.2020.09.057 33186530 PMC7781065

[B22] Guillemot F. Hassan B. A. (2017). Beyond proneural: Emerging functions and regulations of proneural proteins. *Curr. Opin. Neurobiol.* 42 93–101. 10.1016/j.conb.2016.11.011 28025176

[B23] Haider B. Duque A. Hasenstaub A. R. McCormick D. A. (2006). Neocortical network activity in vivo is generated through a dynamic balance of excitation and inhibition. *J. Neurosci.* 26 4535–4545. 10.1523/JNEUROSCI.5297-05.2006 16641233 PMC6674060

[B24] Han S. Dennis D. J. Balakrishnan A. Dixit R. Britz O. Zinyk D. (2018). A non-canonical role for the proneural gene Neurog1 as a negative regulator of neocortical neurogenesis. *Development* 145:dev157719. 10.1242/dev.157719 30201687 PMC6198467

[B25] He L. Yu K. Lu F. Wang J. Wu L. N. Zhao C. (2018). Transcriptional regulator ZEB2 is essential for Bergmann glia development. *J. Neurosci.* 38 1575–1587. 10.1523/JNEUROSCI.2674-17.2018 29326173 PMC5815355

[B26] Hensch T. K. (2005). Critical period plasticity in local cortical circuits. *Nat. Rev. Neurosci.* 6 877–888. 10.1038/nrn1787 16261181

[B27] Hevner R. F. (2019). Intermediate progenitors and Tbr2 in cortical development. *J. Anat.* 235 616–625. 10.1111/joa.12939 30677129 PMC6656625

[B28] Huang Z. J. Paul A. (2019). The diversity of GABAergic neurons and neural communication elements. *Nat. Rev. Neurosci.* 20 563–572. 10.1038/s41583-019-0195-4 31222186 PMC8796706

[B29] Ivanovski I. Djuric O. Caraffi S. G. Santodirocco D. Pollazzon M. Rosato S. (2018). Phenotype and genotype of 87 patients with Mowat-Wilson syndrome and recommendations for care. *Genet. Med.* 20 965–975. 10.1038/gim.2017.221 29300384

[B30] Jang S. S. Kim S. Y. Kim H. Hwang H. Chae J. H. Kim K. J. (2019). Diagnostic yield of epilepsy panel testing in patients with seizure onset within the first year of life. *Front. Neurol.* 10:988. 10.3389/fneur.2019.00988 31572294 PMC6753218

[B31] Kepecs A. Fishell G. (2014). Interneuron cell types are fit to function. *Nature* 505 318–326. 10.1038/nature12983 24429630 PMC4349583

[B32] Kessaris N. Magno L. Rubin A. N. Oliveira M. G. (2014). Genetic programs controlling cortical interneuron fate. *Curr. Opin. Neurobiol.* 26 79–87. 10.1016/j.conb.2013.12.012 24440413 PMC4082532

[B33] Khoshkhoo S. Vogt D. Sohal V. S. (2017). Dynamic, cell-type-specific roles for GABAergic interneurons in a mouse model of optogenetically inducible seizures. *Neuron* 93 291–298. 10.1016/j.neuron.2016.11.043 28041880 PMC5268075

[B34] Kim D. W. Liu K. Wang Z. Q. Zhang Y. S. Bathini A. Brown M. P. (2021). Gene regulatory networks controlling differentiation, survival, and diversification of hypothalamic Lhx6-expressing GABAergic neurons. *Commun. Biol.* 4:95. 10.1038/s42003-020-01616-7 33479483 PMC7820013

[B35] Kim W. Y. (2012). NeuroD1 is an upstream regulator of NSCL1. *Biochem. Biophys. Res. Commun.* 419 27–31. 10.1016/j.bbrc.2012.01.100 22310718

[B36] Kobeissy F. H. Hansen K. Neumann M. Fu S. Jin K. Liu J. (2016). Deciphering the role of Emx1 in neurogenesis: A neuroproteomics approach. *Front. Mol. Neurosci.* 9:98. 10.3389/fnmol.2016.00098 27799894 PMC5065984

[B37] Kou B. Liu W. Tang X. Kou Q. (2018). HMGA2 facilitates epithelial-mesenchymal transition in renal cell carcinoma by regulating the TGF-beta/Smad2 signaling pathway. *Oncol. Rep.* 39 101–108. 10.3892/or.2017.6091 29138866 PMC5783590

[B38] Lim L. Marin O. (2018). More than one way to induce a neuron. *Nature* 557 316–317. 10.1038/d41586-018-04978-5 29752451

[B39] Lim L. Mi D. Llorca A. Marin O. (2018). Development and functional diversification of cortical interneurons. *Neuron* 100 294–313. 10.1016/j.neuron.2018.10.009 30359598 PMC6290988

[B40] Liodis P. Denaxa M. Grigoriou M. Akufo-Addo C. Yanagawa Y. Pachnis V. (2007). Lhx6 activity is required for the normal migration and specification of cortical interneuron subtypes. *J. Neurosci.* 27 3078–3089. 10.1523/JNEUROSCI.3055-06.2007 17376969 PMC6672459

[B41] Liu F. Zhang Y. Chen F. Yuan J. Li S. Han S. (2021). Neurog2 directly converts astrocytes into functional neurons in midbrain and spinal cord. *Cell Death Dis.* 12:225. 10.1038/s41419-021-03498-x 33649354 PMC7921562

[B42] Love M. I. Huber W. Anders S. (2014). Moderated estimation of fold change and dispersion for RNA-seq data with DESeq2. *Genome Biol.* 15:550. 10.1186/s13059-014-0550-8 25516281 PMC4302049

[B43] Lv X. Ren S. Q. Zhang X. J. Shen Z. Ghosh T. Xianyu A. (2019). TBR2 coordinates neurogenesis expansion and precise microcircuit organization *via* Protocadherin 19 in the mammalian cortex. *Nat. Commun.* 10:3946. 10.1038/s41467-019-11854-x 31477701 PMC6718393

[B44] Matsuda T. Irie T. Katsurabayashi S. Hayashi Y. Nagai T. Hamazaki N. (2019). Pioneer factor NeuroD1 rearranges transcriptional and epigenetic profiles to execute microglia-neuron conversion. *Neuron* 101 472.e–485.e. 10.1016/j.neuron.2018.12.010 30638745

[B45] McKinsey G. L. Lindtner S. Trzcinski B. Visel A. Pennacchio L. A. Huylebroeck D. (2013). Dlx1&2-dependent expression of Zfhx1b (Sip1, Zeb2) regulates the fate switch between cortical and striatal interneurons. *Neuron* 77 83–98. 10.1016/j.neuron.2012.11.035 23312518 PMC3547499

[B46] Mi H. Muruganujan A. Casagrande J. T. Thomas P. D. (2013). Large-scale gene function analysis with the PANTHER classification system. *Nat. Protoc.* 8 1551–1566. 10.1038/nprot.2013.092 23868073 PMC6519453

[B47] Miller J. A. Ding S. L. Sunkin S. M. Smith K. A. Ng L. Szafer A. (2014). Transcriptional landscape of the prenatal human brain. *Nature* 508 199–206. 10.1038/nature13185 24695229 PMC4105188

[B48] Miquelajauregui A. Van de Putte T. Polyakov A. Nityanandam A. Boppana S. Seuntjens E. (2007). Smad-interacting protein-1 (Zfhx1b) acts upstream of Wnt signaling in the mouse hippocampus and controls its formation. *Proc. Natl. Acad. Sci. U.S.A.* 104 12919–12924. 10.1073/pnas.0609863104 17644613 PMC1929013

[B49] Miyoshi G. Fishell G. (2011). GABAergic interneuron lineages selectively sort into specific cortical layers during early postnatal development. *Cereb. Cortex* 21 845–852. 10.1093/cercor/bhq155 20732898 PMC3059886

[B50] Mowat D. R. Croaker G. D. Cass D. T. Kerr B. A. Chaitow J. Ades L. C. (1998). Hirschsprung disease, microcephaly, mental retardation, and characteristic facial features: Delineation of a new syndrome and identification of a locus at chromosome 2q22-q23. *J. Med. Genet.* 35 617–623. 10.1136/jmg.35.8.617 9719364 PMC1051383

[B51] Nishizaki Y. Takagi T. Matsui F. Higashi Y. (2014). SIP1 expression patterns in brain investigated by generating a SIP1-EGFP reporter knock-in mouse. *Genesis* 52 56–67. 10.1002/dvg.22726 24243579

[B52] Nouri P. Gotz S. Rauser B. Irmler M. Peng C. Trumbach D. (2020). Dose-dependent and subset-specific regulation of midbrain dopaminergic neuron differentiation by LEF1-mediated WNT1/b-catenin signaling. *Front. Cell Dev. Biol.* 8:587778. 10.3389/fcell.2020.587778 33195246 PMC7649324

[B53] Nowakowski T. J. Bhaduri A. Pollen A. A. Alvarado B. Mostajo-Radji M. A. Di Lullo E. (2017). Spatiotemporal gene expression trajectories reveal developmental hierarchies of the human cortex. *Science* 358 1318–1323. 10.1126/science.aap8809 29217575 PMC5991609

[B54] Pastor W. A. Liu W. Chen D. Ho J. Kim R. Hunt T. J. (2018). TFAP2C regulates transcription in human naive pluripotency by opening enhancers. *Nat. Cell Biol.* 20 553–564. 10.1038/s41556-018-0089-0 29695788 PMC5926822

[B55] Paz J. T. Huguenard J. R. (2015). Microcircuits and their interactions in epilepsy: Is the focus out of focus? *Nat. Neurosci.* 18 351–359. 10.1038/nn.3950 25710837 PMC4561622

[B56] Pfisterer U. Petukhov V. Demharter S. Meichsner J. Thompson J. J. Batiuk M. Y. (2020). Identification of epilepsy-associated neuronal subtypes and gene expression underlying epileptogenesis. *Nat. Commun.* 11:5038. 10.1038/s41467-020-18752-7 33028830 PMC7541486

[B57] Richards S. Aziz N. Bale S. Bick D. Das S. Gastier-Foster J. (2015). Standards and guidelines for the interpretation of sequence variants: A joint consensus recommendation of the American college of medical genetics and genomics and the association for molecular pathology. *Genet. Med.* 17 405–424. 10.1038/gim.2015.30 25741868 PMC4544753

[B58] Roybon L. Mastracci T. L. Ribeiro D. Sussel L. Brundin P. Li J. Y. (2010). GABAergic differentiation induced by Mash1 is compromised by the bHLH proteins Neurogenin2, NeuroD1, and NeuroD2. *Cereb. Cortex* 20 1234–1244. 10.1093/cercor/bhp187 19767311

[B59] Schurch N. J. Schofield P. Gierlinski M. Cole C. Sherstnev A. Singh V. (2016). How many biological replicates are needed in an RNA-seq experiment and which differential expression tool should you use? *RNA* 22 839–851. 10.1261/rna.053959.115 27022035 PMC4878611

[B60] Schuster J. Laan L. Klar J. Jin Z. Huss M. Korol S. (2019a). Transcriptomes of Dravet syndrome iPSC derived GABAergic cells reveal dysregulated pathways for chromatin remodeling and neurodevelopment. *Neurobiol. Dis.* 132:104583. 10.1016/j.nbd.2019.104583 31445158

[B61] Schuster J. Sobol M. Fatima A. Khalfallah A. Laan L. Anderlid B. M. (2019b). Mowat-Wilson syndrome: Generation of two human iPS cell lines (UUIGPi004A and UUIGPi005A) from siblings with a truncating ZEB2 gene variant. *Stem Cell Res.* 39:101518. 10.1016/j.scr.2019.101518 31376723

[B62] Schuurmans C. Armant O. Nieto M. Stenman J. M. Britz O. Klenin N. (2004). Sequential phases of cortical specification involve neurogenin-dependent and -independent pathways. *EMBO J.* 23 2892–2902. 10.1038/sj.emboj.7600278 15229646 PMC514942

[B63] Sobol M. Raykova D. Cavelier L. Khalfallah A. Schuster J. Dahl N. (2015). Methods of reprogramming to induced pluripotent stem cell associated with chromosomal integrity and delineation of a chromosome 5q candidate Region for growth advantage. *Stem Cells Dev.* 24 2032–2040. 10.1089/scd.2015.0061 25867454

[B64] Stuart T. Butler A. Hoffman P. Hafemeister C. Papalexi E. Mauck W. M. III (2019). Comprehensive integration of single-cell data. *Cell* 177 1888.e–1902.e. 10.1016/j.cell.2019.05.031 31178118 PMC6687398

[B65] Sunkin S. M. Ng L. Lau C. Dolbeare T. Gilbert T. L. Thompson C. L. (2013). Allen brain atlas: An integrated spatio-temporal portal for exploring the central nervous system. *Nucleic Acids Res.* 41 D996–D1008. 10.1093/nar/gks1042 23193282 PMC3531093

[B66] Takagi T. Nishizaki Y. Matsui F. Wakamatsu N. Higashi Y. (2015). De novo inbred heterozygous Zeb2/Sip1 mutant mice uniquely generated by germ-line conditional knockout exhibit craniofacial, callosal and behavioral defects associated with Mowat-Wilson syndrome. *Hum. Mol. Genet.* 24 6390–6402. 10.1093/hmg/ddv350 26319231

[B67] Theveneau E. Duband J. L. Altabef M. (2007). Ets-1 confers cranial features on neural crest delamination. *PLoS One* 2:e1142. 10.1371/journal.pone.0001142 17987123 PMC2043494

[B68] Thuault S. Tan E. J. Peinado H. Cano A. Heldin C. H. Moustakas A. (2008). HMGA2 and Smads co-regulate SNAIL1 expression during induction of epithelial-to-mesenchymal transition. *J. Biol. Chem.* 283 33437–33446. 10.1074/jbc.M802016200 18832382 PMC2662269

[B69] Tutukova S. Tarabykin V. Hernandez-Miranda L. R. (2021). The role of neurod genes in brain development, function, and disease. *Front. Mol. Neurosci.* 14:662774. 10.3389/fnmol.2021.662774 34177462 PMC8221396

[B70] Van de Putte T. Francis A. Nelles L. van Grunsven L. A. Huylebroeck D. (2007). Neural crest-specific removal of Zfhx1b in mouse leads to a wide range of neurocristopathies reminiscent of Mowat-Wilson syndrome. *Hum. Mol. Genet.* 16 1423–1436. 10.1093/hmg/ddm093 17478475

[B71] Van de Putte T. Maruhashi M. Francis A. Nelles L. Kondoh H. Huylebroeck D. (2003). Mice lacking ZFHX1B, the gene that codes for Smad-interacting protein-1, reveal a role for multiple neural crest cell defects in the etiology of Hirschsprung disease-mental retardation syndrome. *Am. J. Hum. Genet.* 72 465–470. 10.1086/346092 12522767 PMC379238

[B72] van den Berghe V. Stappers E. Vandesande B. Dimidschstein J. Kroes R. Francis A. (2013). Directed migration of cortical interneurons depends on the cell-autonomous action of Sip1. *Neuron* 77 70–82. 10.1016/j.neuron.2012.11.009 23312517

[B73] Wang C. Kam R. K. Shi W. Xia Y. Chen X. Cao Y. (2015). The proto-oncogene transcription factor Ets1 regulates neural crest development through histone deacetylase 1 to mediate output of bone morphogenetic protein signaling. *J. Biol. Chem.* 290 21925–21938. 10.1074/jbc.M115.644864 26198637 PMC4571947

[B74] Wang C. Lee J. E. Lai B. Macfarlan T. S. Xu S. Zhuang L. (2016). Enhancer priming by H3K4 methyltransferase MLL4 controls cell fate transition. *Proc. Natl. Acad. Sci. U.S.A.* 113 11871–11876. 10.1073/pnas.1606857113 27698142 PMC5081576

[B75] Wang J. Lin Z. J. Liu L. Xu H. Q. Shi Y. W. Yi Y. H. (2017). Epilepsy-associated genes. *Seizure* 44 11–20. 10.1016/j.seizure.2016.11.030 28007376

[B76] Wang Y. Xu C. Xu Z. Ji C. Liang J. Wang Y. (2017). Depolarized GABAergic signaling in subicular microcircuits mediates generalized seizure in temporal lobe epilepsy. *Neuron* 95 92.e–105.e. 10.1016/j.neuron.2017.06.004 28648501

[B77] Yalim-Camci I. Balcik-Ercin P. Cetin M. Odabas G. Tokay N. Sayan A. E. (2019). ETS1 is coexpressed with ZEB2 and mediates ZEB2-induced epithelial-mesenchymal transition in human tumors. *Mol. Carcinog.* 58 1068–1081. 10.1002/mc.22994 30790340

[B78] Yang Y. Shen W. Ni Y. Su Y. Yang Z. Zhao C. (2017). Impaired interneuron development after foxg1 disruption. *Cereb. Cortex* 27 793–808. 10.1093/cercor/bhv297 26620267

[B79] Zweier C. Albrecht B. Mitulla B. Behrens R. Beese M. Gillessen-Kaesbach G. (2002). “Mowat-Wilson” syndrome with and without hirschsprung disease is a distinct, recognizable multiple congenital anomalies-mental retardation syndrome caused by mutations in the zinc finger homeo box 1B gene. *Am. J. Med. Genet.* 108 177–181. 10.1002/ajmg.10226 11891681

