# Supplementary figures and images for: ZEB2 haploinsufficient Mowat-Wilson syndrome induced pluripotent stem cells show disrupted GABAergic transcriptional regulation and function
Source: Front Mol Neurosci. 2022 Oct 24;15:988993. doi: 10.3389/fnmol.2022.988993 (PMC9637781; doi:10.3389/fnmol.2022.988993)

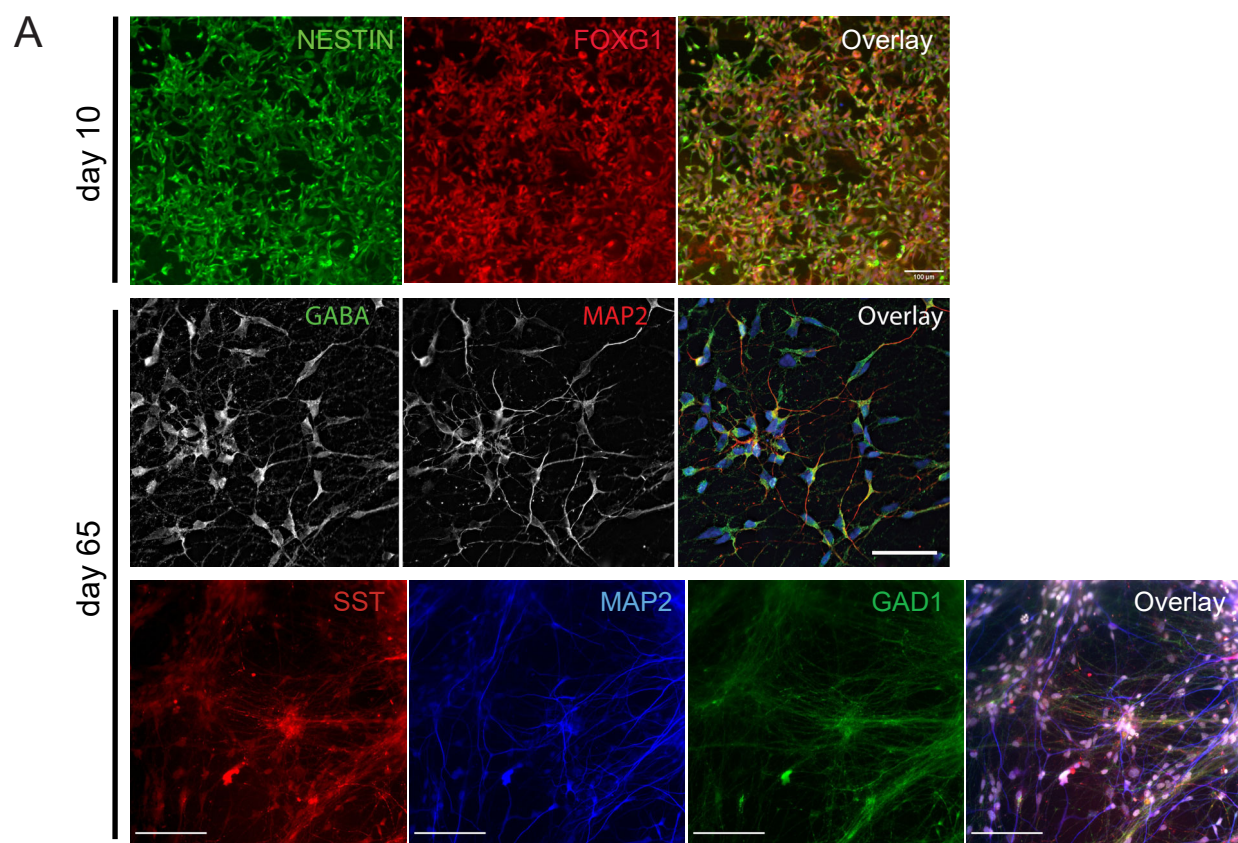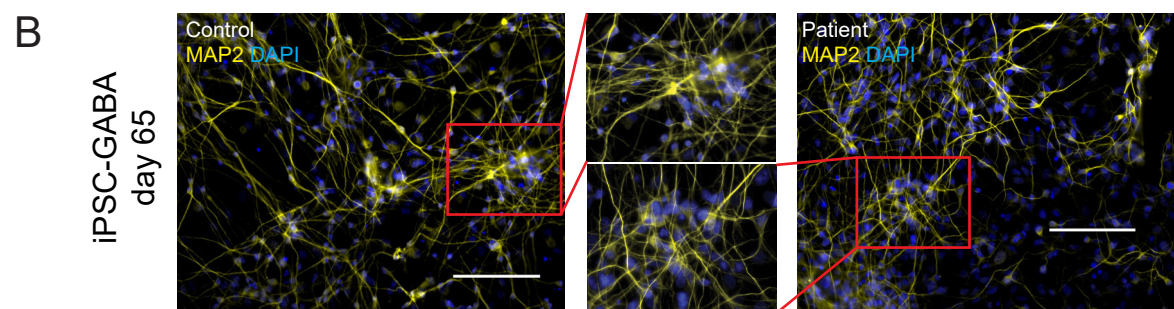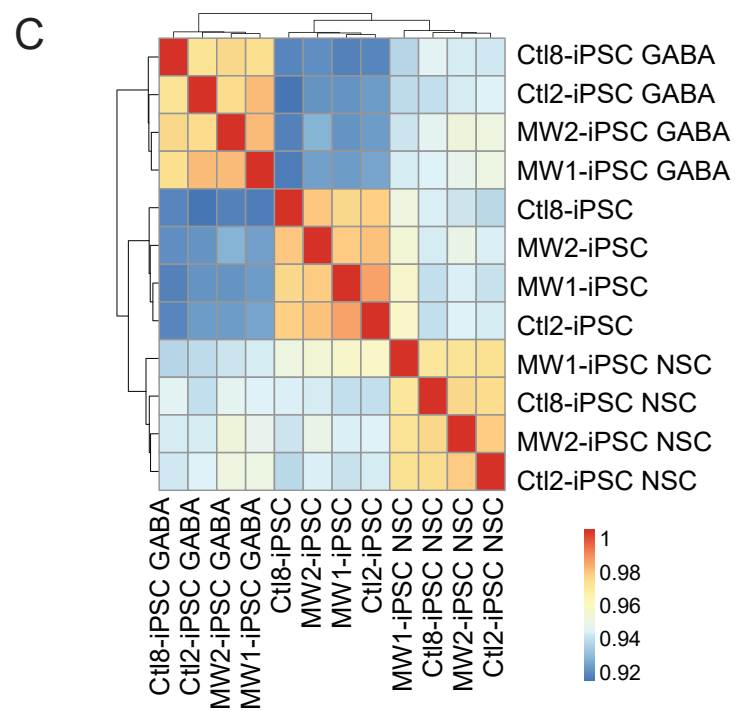

Supplement: Supplementary file 1 [file Image_1.pdf]

A

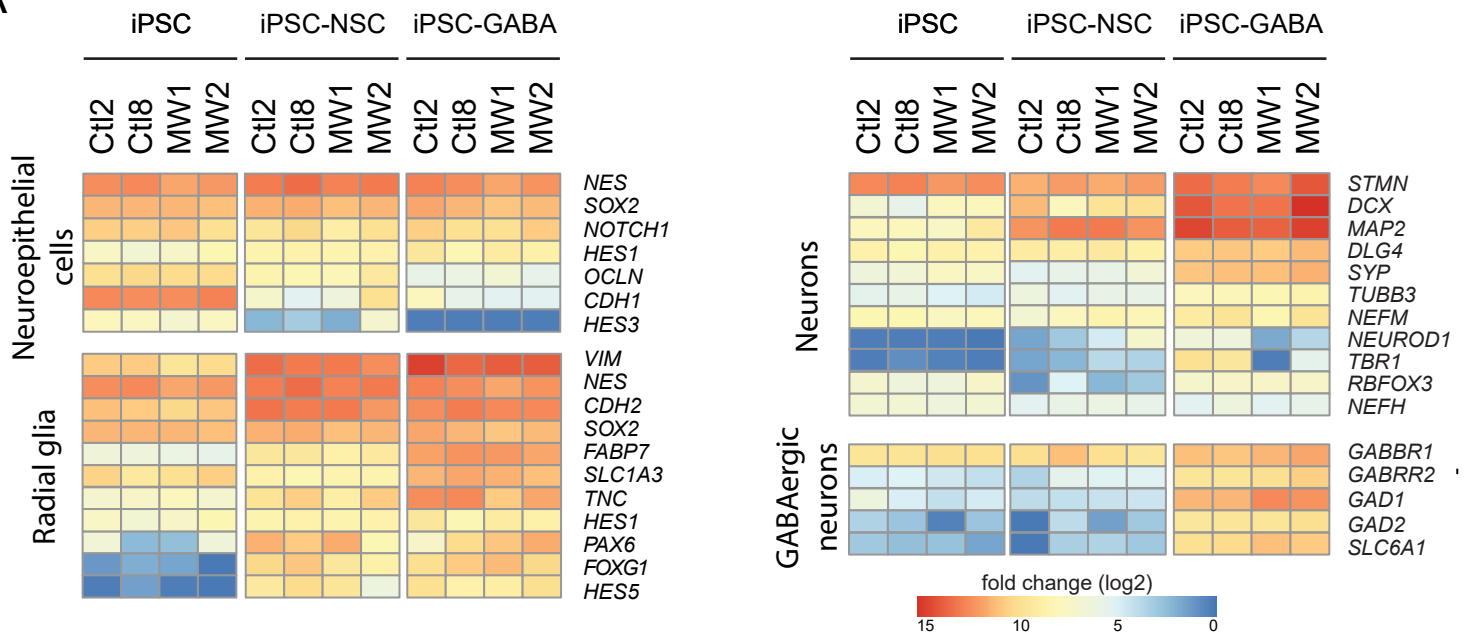

B

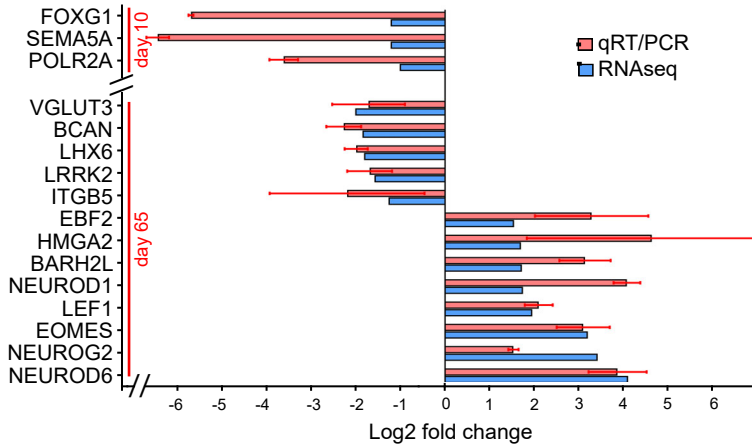

C

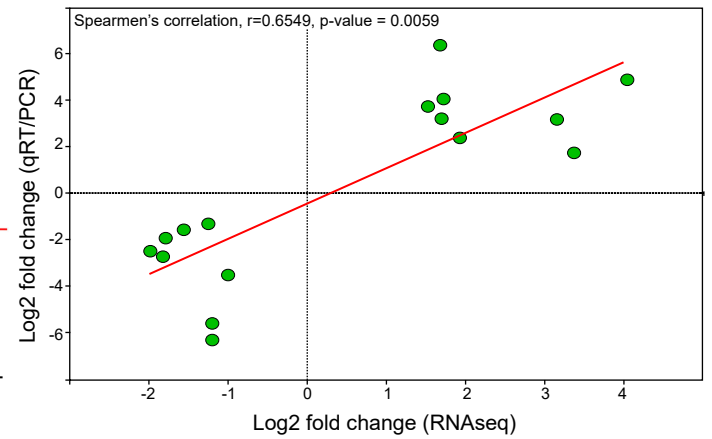

D

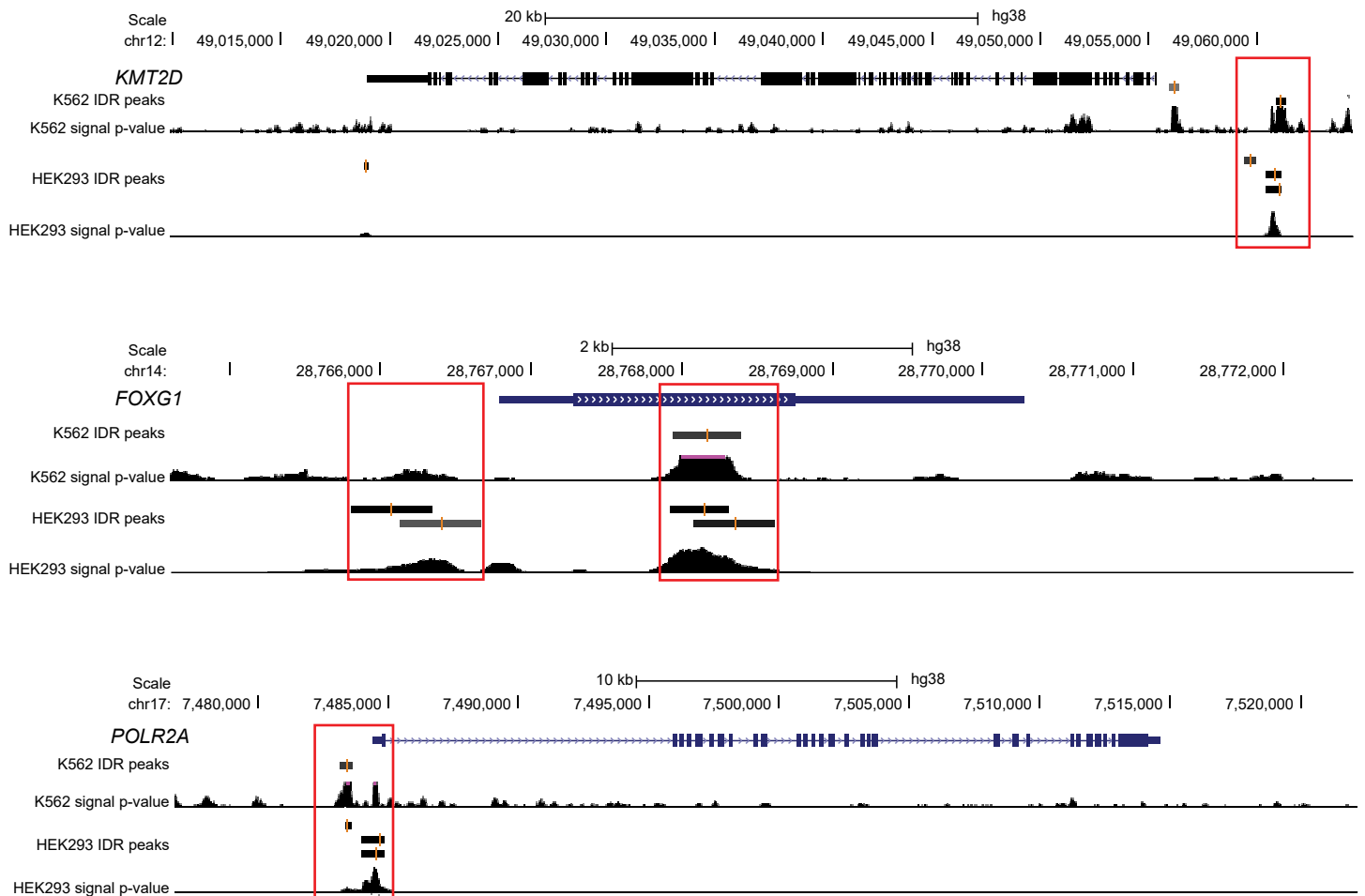

Supplement: Supplementary file 2 [file Image_2.pdf]
