# Supplementary material for: ZEB2 haploinsufficient Mowat-Wilson syndrome induced pluripotent stem cells show disrupted GABAergic transcriptional regulation and function
Source: Front Mol Neurosci. 2022 Oct 24;15:988993. doi: 10.3389/fnmol.2022.988993 (PMC9637781; doi:10.3389/fnmol.2022.988993)
Supplement: Supplementary file 3 [file Image_3.pdf]

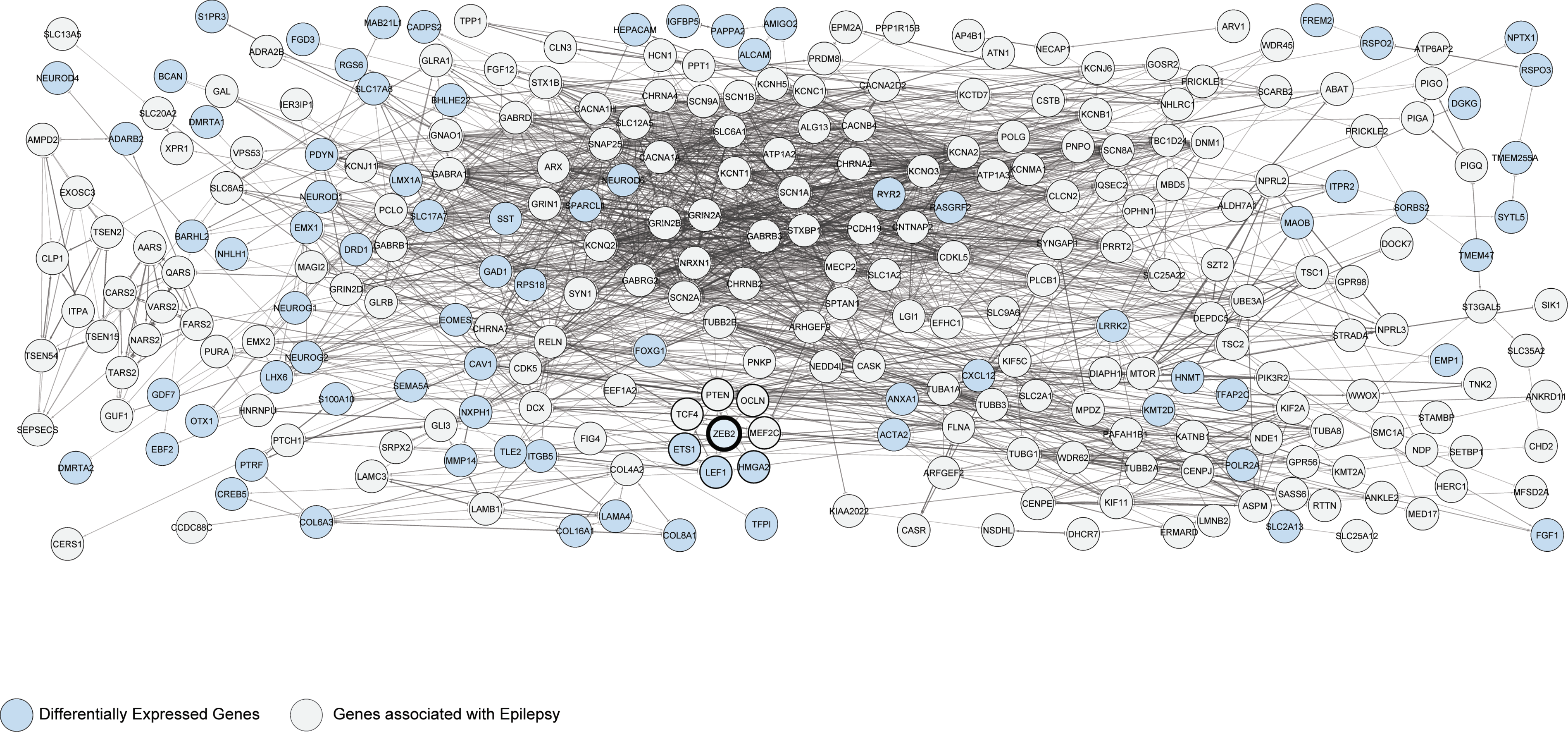
S1PR3
MAB21L1
CADPS2
TPP1
HEPACAM
IGFBP5
PAPPA2
AMIGO2
EPM2A
PPP1R15B
AP4B1
ARV1
FREM2
NPTX1
SLC13A5
ADRA2B
FGD3
CLN3
HCN1
ALCAM
PRDM8
ATN1
NECAP1
WDR45
RSPO2
ATP6AP2
RSPO3
NEUROD4
RGS6
GLRA1
FGF12
PPT1
KCNH5
KCNC1
CACNA2D2
KCNJ6
GOSR2
PRICKLE1
SCARB2
ABAT
PIGO
BCAN
GAL
SLC17A8
STX1B
CHRNA4
SCN9A
SCN1B
KCTD7
CSTB
NHLRC1
DGKG
IER3IP1
BHLHE22
CACNA1H
KCNB1
PIGA
SLC20A2
DMRTA1
GABRD
SLC12A5
ALG13
CACNB4
PRICKLE2
AMPD2
ADARB2
XPR1
VPS53
GNAO1
SNAP25
SLC6A1
POLG
TBC1D24
DNM1
PDYN
KCNJ11
CACNA1A
ATP1A2
KCNA2
PNPO
SCN8A
TMEM255A
PIGQ
EXOSC3
LMX1A
GABRA1
ARX
NEURODG
KCNT1
CHRNA2
KCNQ3
ATP1A3
KCNMA1
MBD5
NPRL2
ITPR2
SLC6A5
NEUROD1
PCLO
SLC17A7
SST
GRIN1
SPARCL1
SCN1A
RYR2
IQSEC2
CLCN2
ALDH7A1
SORBS2
TSEN2
EMX1
GRIN2B
GRIN2A
RASGRF2
OPHN1
MAOB
SYTL5
AARS
BARHL2
DRD1
GABRB1
KCNQ2
GABRB3
STXBP1
PCDH19
CNTNAP2
SYNGAP1
PRRT2
DOCK7
CLP1
NHLH1
MAGI2
NRXN1
MECP2
CDKL5
TMEM47
QARS
GAD1
SLC1A2
SZT2
TSC1
CARS2
GRIN2D
RPS18
GABRG2
CHRNB2
PLCB1
SLC25A22
GPR98
ITPA
VARS2
GLRB
SYN1
SCN2A
SPTAN1
UBE3A
ST3GAL5
SIK1
NEUROG1
LGI1
EFHC1
SLC9A6
DEPDC5
TSEN15
FARS2
EOMES
CHRNA7
TUBB2B
ARHGEF9
LRRK2
NARS2
STRADA
NPRL3
TSEN54
EMX2
RELN
FOXG1
TSC2
PURA
NEUROG2
CAV1
CDK5
PNKP
NEDD4L
CASK
KIF5C
SLC35A2
EMP1
TARS2
LHX6
SEMA5A
CXCL12
DIAPH1
MTOR
HNMT
PIK3R2
TNK2
GDF7
S100A10
EEF1A2
PTEN
OCLN
ANXA1
TUBA1A
TFAP2C
WWOX
ANKRD11
GUF1
HNRNPU
NXPH1
DCX
TCF4
TUBB3
SLC2A1
MPDZ
KMT2D
KIF2A
STAMBP
SEPSECS
OTX1
GLI3
ZEB2
MEF2C
FLNA
PAFAH1B1
KATNB1
NDE1
TUBA8
SMC1A
PTCH1
FIG4
ACTA2
NDP
SETBP1
CHD2
EBF2
SRPX2
TLE2
ITGB5
ETS1
TUBG1
WDR62
GPR56
DMRTA2
PTRF
MMP14
COL4A2
HMGA2
LEF1
ARFGEF2
TUBB2A
CENPJ
POLR2A
KMT2A
LAMC3
HERC1
CREB5
SASS6
RTTN
ANKLE2
MFSD2A
LAMB1
CENPE
KIF11
ASPM
KIAA2022
LAMA4
COL6A3
LMNB2
MED17
CCDC88C
TFPI
CASR
NSDHL
DHCR7
ERMARD
SLC2A13
SLC25A12
FGF1
COL16A1
COL8A1
CERS1
Differentially Expressed Genes
Genes associated with Epilepsy
